# Phytochemical and Bioactivity Evaluation of Bee Pollen and Androecia of *Castanea*, *Salix*, and *Quercus* Species

**DOI:** 10.3390/antiox14010040

**Published:** 2024-12-31

**Authors:** Nisa Beril Sen, Irena Vovk, Hasan Kırmızıbekmez, Etil Guzelmeric

**Affiliations:** 1 Department of Pharmacognosy, Faculty of Pharmacy, Yeditepe University, Kayisdagi Cad., Atasehir, 34755 Istanbul, Türkiye; nisaberil.sen@yeditepe.edu.tr (N.B.S.); hkirmizibekmez@yeditepe.edu.tr (H.K.); 2 Laboratory for Food Chemistry, National Institute of Chemistry, Hajdrihova 19, SI-1000 Ljubljana, Slovenia

**Keywords:** bee pollen, *N*^1^,*N*^5^,*N*^10^-tricaffeoylspermidine, HPTLC, UPLC, HPTLC-effect-directed analyses, antioxidant activity, xanthine oxidase inhibitory activity, chemical profiling, image analysis

## Abstract

Qualitative and quantitative differences in the chemical composition between bee pollen originated from *Castanea sativa* (Türkiye and Slovenia), *Salix* spp. (Türkiye and Slovenia), and *Quercus* spp. (Türkiye) and androecia of *Castanea sativa*, *Salix alba*, and *Quercus pubescens* (apetalous trees) were evaluated for the first time by new high-performance thin-layer chromatography (HPTLC) and ultra-performance liquid chromatography (UPLC) methods using marker compounds. *N*^1^,*N*^5^,*N*^10^-tricaffeoylspermidine was isolated, and its structure was elucidated by nuclear magnetic resonance (NMR) and high-resolution mass spectrometry (HRMS). It was the main and the marker compound common to bee pollen (≈3–41 mg/g) and androecia (≈3–6 mg/g) samples. To the best of our knowledge, this is the first report of the identification of *N*^1^,*N*^5^,*N*^10^-tricaffeoylspermidine in bee pollen originated from *Salix* spp. and androecia of *C. sativa*, *S. alba*, and *Q. pubescens*. The botanical origins of bee pollen were determined via phytochemical profiling using HPTLC-image analyses showing that bee pollen from the same botanical source had almost identical profiles regardless of collection location, geographical differences, and the bee race. *In vitro* tests and HPTLC-effect-directed analyses (EDAs) were performed to assess antioxidant and xanthine oxidase (XO) inhibitory activities of bee pollen, androecia, and *N*^1^,*N*^5^,*N*^10^-tricaffeoylspermidine. HPTLC-EDA combined with image analyses was used for comparing the activities of bee pollen, androecia, *N*^1^,*N*^5^,*N*^10^-tricaffeoylspermidine, and also other marker compounds (quercetin, myricitrin, hyperoside, quercitrin, isoquercitrin, and rutin). The remarkable bioactivity of *N*^1^,*N*^5^,*N*^10^-tricaffeoylspermidine was for the first time evaluated by HPTLC-EDA and *in vitro* tests. This is the first study performing HPTLC-XO inhibitory activity analyses on the HPTLC NH_2_ F_254S_ plates. Further bioactivity studies on botanically and chemically well-characterized bee pollen samples are needed to aid in the use of bee pollen-containing supplements in the prevention and treatment of diseases.

## 1. Introduction

A complete flower consists of four main parts: green-colored sepals, colorful petals, and male (androecium) and female (gynoecium) reproductive organs. The apetalous flower is a type of incomplete flower without petals that serves as a valuable food source for bees as bees directly come into contact with the anthers (a part of androecium) containing pollen sacs. When electrically charged bees come close to these flowers, their bodies are covered by pollen grains due to the electrostatic field. These pollen grains are converted into pellets by honeybees using their saliva, flower nectar, honey, etc. The final product is known as bee pollen. During this transformation, bee pollen grains may have morphologically and chemically different characteristic features from flower pollen [1]. The main bioactive compounds of bee pollen are phenolic acids (e.g., chlorogenic acid and *p*-coumaric acid), flavonoids (e.g., quercetin, kaempferol, and their glycoside derivatives), and phenolamides, which are formed by conjunction of hydroxycinnamic acid derivatives (e.g., caffeic acid, *p*-coumaric acid, and ferulic acid) with putrescine, spermidine, or spermine [2,3,4,5,6]. The presence and quantity of these compounds strongly depend on the botanical source of the bee pollen. It is, therefore, relevant to characterize the main chemical composition of bee pollen, which is linked to its therapeutic properties such as antioxidant, anti-inflammatory, anticariogenic, antibacterial, hepatoprotective, anti-atherosclerotic, and immune enhancing potential [2].

A fraction with a molecular weight less than 1000 obtained from an aqueous extract of *Cistus ladaniferus* bee pollen was found to be responsible for stimulating bone formation and inhibiting osteoclastic bone resorption [7]. The lack of knowledge of the chemical components responsible for the targeted bioactivity causes difficulties in the application of bee pollen supplements in phytotherapy. Standardization over bioactive component(s) is a key parameter for achieving reproducible pharmacological activity. Another challenge for the therapeutic use of food supplements containing bee pollen is that the botanical origin of bee pollen is not well defined. This is because palynological analysis applied to determine the botanical source of bee pollen using a microscope is sometimes insufficient for the identification of species with similar pollen grains; in this case, identification is accomplished either by giving the genus name (*Cistus* spp., *Papaver* spp., or *Salix* spp.) or the family name (Asteraceae, Brassicaceae, Boraginaceae, etc.). There are many species in a genus or a family; therefore, their chemical composition will be different. One study showed that the anti-inflammatory activity of an ethanolic extract of *Cistus* spp. originated bee pollen collected in Spain contained quercetin, kaempferol, and their derivatives [8]. In Spain, various *Cistus* spp. are grown such as *Cistus ladanifer* L., *Cistus albidus* L., *Cistus salviifolius* L., and *Cistus clusii* and their chemical composition may differ from each other [9]. Thus, there is a need for alternative methods that can be used for identifying the botanical source of bee pollen.

High-performance thin-layer chromatography (HPTLC) enables simultaneous analysis of bee pollen and its flower source under the same conditions, leading to the determination of the botanical origin of bee pollen. In a recent study, HPTLC profiling of flower and bee pollen of *Hedera helix* led to the determination of the botanical origin of bee pollen samples. Additionally, when hydroalcoholic bee pollen extracts were standardized using common markers such as quercetin-3-*O*-β-glucopyranosyl-(1→2)-β-galactopyranoside, platanoside, and afzelin found both in the *H. helix* flower and bee pollen, they exhibited antioxidant and xanthine oxidase (XO) inhibitory activities [10]. Further bioactivity studies on botanically and chemically well-characterized bee pollen samples are needed to aid in the use of bee pollen-containing supplements in the prevention and treatment of diseases.

*Castanea sativa* (sweet chestnut), *Salix* spp. (willow), and *Quercus* spp. (oak) are examples of trees having apetalous flowers. Their inflorescence, known as catkins, contains male flowers responsible for releasing pollen grains. In Türkiye and Slovenia, these trees grow all over the county, providing distinctive sources to honeybees for bee pollen production. Apparently, there are only a few phytochemical and bioactivity studies about *Castanea sativa* [11,12], *Salix* spp. [13,14,15,16,17,18], and *Quercus* spp. flower pollen [19]. The methanol extract of *C. sativa* flower pollen collected from nine populations showed high antibacterial activity, especially against *Micrococcus luteus*, methicillin resistant *Staphylococcus aureus*, and *Streptococcus aureus* [11]. Among those extracts, the one with the highest total phenolic content (TPC) value showed the highest 2,2-diphenyl-1-picrylhydrazyl (DPPH) radical scavenging activity [11]. Anti-urease activity was reported for aqueous and methanolic extracts of *C. sativa* blossoms in which rutin was found to be the dominant phenolic compound [12]. There are no bioactivity studies for *S. alba* flowers; although, some other *Salix* species displayed antioxidant, hepatoprotective, neuroprotective, antibacterial, and anticancer activities. *Salix* spp. flowers’ extracts in methanol (*S. aegyptiaca*, *S. matsudana*, *S. babylonica*, *S. excelsa*, and *S. acmophylla*), ethanol (*S. caprea* and *S. aegyptiaca*), cyclohexane (*S. aegyptiaca*), butanol (*S. aegyptiaca*), and water (*S. aegyptiaca*) showed antioxidant activity by various assays such as DPPH, superoxide hydrogen peroxide, and nitric oxide scavenging assays [14,15]. The ethanol extract of *S. caprea* flowers significantly suppressed hepatic lipid peroxidation (LPO) which indicated its hepatoprotective effect [14]. The acetone-soluble fraction of *S. caprea* flowers was shown to be an effective antioxidant and chemopreventive agent against phorbol ester-induced tumor promotion *in vivo* [13]. The anti-inflammatory and antimicrobial activities of the methanolic extract of *S. tetrasperma* flowers, with dominant rutin, kaempferide 3-*O*-glucoside, trichocarposide, coumaroylquinic acid, and salicin were investigated, resulting in the extract significantly reducing the expression of several pro-inflammatory biomarkers in brainstem and sciatic nerve tissue, as well inhibiting *P. aeruginosa* PAO1 growth and biofilm formation [17,18]. In addition, the ethanol extract of *S. subserrata* flowers significantly reduced the elevated serum levels of intracellular liver enzymes as well as liver biomarkers in carbon tetrachloride (CCl_4_)-induced liver damage *in vivo*, demonstrating its hepatoprotective effect [16]. There is only one phytochemical study on *Quercus* spp. flower pollen. Accordingly, hydroxycinnamic acid amides were isolated from *Quercus dentata* flower pollen, and their structures were elucidated as *N*^1^,*N*^5^,*N*^10^-tricaffeoylspermidine, *N*^1^-*p*-coumaroyl-*N*^5^,*N*^10^-dicaffeoylspermidine, *N*^1^,*N*^10^-di-*p*-coumaroyl-*N*^5^-caffeoylspermidine, and *N*^1^,*N*^5^,*N*^10^-tri-*p*-coumaroylspermidine. In view of the chemical composition and the health beneficial effects of the flowers of these species’ bee pollen, which is botanically derived from these species, has recently been the focus of scientific studies.

Uric acid is formed by an enzyme called xanthine oxidase (XO) by catalyzing the oxidation of hypoxanthine to xanthine to uric acid. During this reaction, reactive oxygen species (ROS) are also produced. Excessive uric acid production, along with ROS, can trigger inflammation that leads to many diseases. Prolonged use of allopurinol, an XO inhibitor, may make individuals more prone to the development of kidney and liver diseases [20]. Therefore, research on compounds derived from natural sources as bee products is of interest to eliminate the risk factors. There are various *in vitro* studies evaluating the xanthine oxidase inhibitory activities of extracts from natural sources. The advantage of HPTLC-effect-directed analysis (HPTLC-EDA) in evaluating XO inhibitory activities of extracts is that, besides providing information on bioactivity, HPTLC-EDA also provides information on the active compound(s) separated on the chromatogram as demonstrated for quercetin-3-*O*-β-glucopyranosyl-(1→2)-β-galactopyranoside and afzelin from bee pollen originated from *H. helix* [10].

The aim of this study was to compare chemical and bioactivity profiles of extracts from *C. sativa*, *Salix* spp., and *Quercus* spp. bee pollen (samples from Türkiye and Slovenia) and androecia, containing anthers composed of pollen sacs of *C. sativa*, *S. alba*, and *Q. pubescens* which were considered to be possible bee pollen sources. The steps of this study were (1) HPTLC profiling of androecia and bee pollen samples; (2) isolation of the marker compound (*N*^1^,*N*^5^,*N*^10^-tricaffeoylspermidine, found in all samples) by column chromatography and structural elucidation by NMR and HRMS; (3) development and validation of the UPLC method to quantify the main components in hydroalcoholic extracts of androecia and bee pollen; (4) evaluation of the antioxidant and XO enzyme inhibition activities of all extracts and *N*^1^,*N*^5^,*N*^10^-tricaffeoylspermidine by spectroscopic methods; (5) evaluation of the antioxidant and XO enzyme inhibition activities of hydroalcoholic extracts from androecia and bee pollen, *N*^1^,*N*^5^,*N*^10^-tricaffeoylspermidine, as well as other marker compounds for androecia and bee pollen by HPTLC-EDA combined with image analysis.

## 2. Materials and Methods

### 2.1. Chemicals

All chemicals used were at least of analytical grade. Methanol (HPLC grade and analytical grade), ethanol, acetonitrile (HPLC grade), ethyl acetate, glacial acetic acid, formic acid (98–100%), and dimethyl sulfoxide (DMSO) were acquired from Sigma-Aldrich (Steinheim, Germany). Xanthine oxidase (XO), 2,2′-azino-bis(3-ethylbenzothiazoline-6-sulfonic acid) diammonium salt (ABTS), 2,2-diphenyl-1-picrylhydrazyl (DPPH·), 2,4,6-tri(2-pyridyl)-s-triazine (TPTZ), sephadex LH-20, and *p*-anisaldehyde were acquired from Sigma-Aldrich (Steinheim, Germany). Sodium acetate, di-sodium hydrogen phosphate heptahydrate, *o*-phosphoric acid (85%), polyethylene glycol 400 (PEG 400), polyamide (for column chromatography), and copper (II) sulfate pentahydrate were from Merck (Darmstadt, Germany). Sodium phosphate monobasic dihydrate, sulfuric acid (95–97%), and hydrochloric acid were from Riedel-de Haen (Seelze, Germany). Ethylenediaminetetraacetic acid (EDTA), iron (III) chloride, and ammonium acetate were obtained from Fluka (Steinheim, Germany). Nitro blue tetrazolium chloride (NBT) was acquired from Cayman Chemical Company (Ann Arbor, MI, USA), and 2-aminoethyl diphenylborinate (NP) was from Alfa Aesar (Karlsruhe, Germany). Standards of rutin, isoquercitrin, hyperoside, myricitrin, quercitrin, quercetin, and trolox ((±)-6-hydroxy-2,5,7,8-tetramethylchromane-2-carboxylic acid) (97%) were purchased from Sigma-Aldrich (Steinheim, Germany). Allopurinol (98%) was acquired from Acros Organics (UK). Neocuproine was obtained from Sigma-Aldrich. Xanthine (99%) was obtained from Alfa Aesar (Kandel, Germany). Distilled water was used for extractions and all analyses except for UPLC analyses, for which ultrapure water was obtained by means of the Simplicity UV purification system (Millipore, Darmstadt, Germany).

### 2.2. Preparation of Standard Solutions

All standard solutions of rutin, isoquercitrin, hyperoside, myricitrin, quercitrin, quercetin, and *N^1^*,*N*^5^,*N*^10^-tricaffeoylspermidine (isolated compound) were prepared in methanol. Standard stock solutions were prepared at concentrations of 200 µg/mL for HPTLC analyses and 350 µg/mL for UPLC analyses. A standard mixture (MIX) for HPTLC analyses was prepared by mixing equal volumes (100 µL) of all seven standard solutions (200 µg/mL). Stock solutions for UPLC analyses (350 µg/mL) were further diluted with methanol to prepare seven working solutions for each standard in a concentration range of 3.5–350 µg/mL. The seven working standard solutions were mixed in the order of the lowest to the highest concentration to prepare standard mixtures for the calibration curves (0.5–50 µg/mL).

### 2.3. Samples

Bee pollen samples were obtained from professional beekeepers who placed their beehives in various regions of Türkiye and Slovenia. Bee pollen samples were encoded after palynological analysis was applied according to the standard methodology [21]. Bee pollen samples were classified according to Barth [22] as dominant pollen (>45%), secondary pollen (15–45%), important minor pollen (3–15%), and minor pollen (<3%). Codes, botanical sources with the proportion of dominant pollen grains, and the collection sites of the bee pollen samples are presented in Table 1.

Bee pollen samples that originated from *Castanea sativa* were encoded as “C” and numbered from 1 to 3, samples originated from *Salix* spp. are encoded as “S” and numbered from 1 to 7, and one sample that originated from *Quercus* spp. is encoded as “Q1” (Table 1). The terminology used in this paper (C-bee pollen, S-bee pollen, and Q-bee pollen) is presented in Table 1.

The *Castanea sativa* Mill. (sweet chestnut), *Salix alba* L. (white willow), and *Quercus pubescens* Willd. (pubescent oak) samples used in this study were selected and collected according to the locations and plant diversity around the beehives. The male flowers (catkins) for *C. sativa* were collected from the forest in İzmit (Türkiye) (40°39′39.0″ N 30°08′01.2″ E) from the same collection site as the bee pollen coded as C1. *S. alba* catkins were collected in Çanakkale (Türkiye) (39°31′33.6″ N 26°16′45.7″ E), closely located to the bee pollen samples encoded as S3. Additionally, *Q. pubescens* is mainly grown in the forests surrounding Ankara [23], where the sample Q1 was obtained. As a representative sample, *Q. pubescens* catkins were collected from Çanakkale (Türkiye) (39°30′35.8″ N 26°17′00.2″ E). These samples were identified by Assoc. Prof. Dr. Etil Guzelmeric. The voucher specimens of *C. sativa* (YEF23009), *S. alba* (YEF24008), and *Q. pubescens* (YEF24009) were deposited at the Herbarium of the Faculty of Pharmacy, Yeditepe University, İstanbul, Türkiye. Finally, plant parts named androecia, containing anthers composed of pollen sacs, were separated from the catkins of *C. sativa*, *S. alba*, and *Q. pubescens.* All androecia samples and bee pollen samples were kept at −20 °C before being used for chemical composition and bioactivity studies.

### 2.4. Preparation of Sample Test Solutions

Bee pollen and androecia samples (5 g) were extracted with 80% ethanol_(aq)_ (50 mL) in an ultrasonic bath (30 min). The solutions obtained were filtered, and the liquid part was evaporated under reduced pressure at 40 °C to obtain hydroalcoholic extracts. Hydroalcoholic extracts (250 mg) were dissolved in methanol (5 mL) using a sonicator and filtered through 0.45 µm hydrophilic-regenerated cellulose (RC) membrane filters (Minisart, Sartorius Stedim Biotech, Goettingen, Germany) to obtain stock sample test solutions (50 mg/mL). Stock sample test solutions diluted with methanol were used for HPTLC analyses (20 mg/mL) and bioactivity analyses (0.25–2.5 mg/mL for free radical-scavenging activity with 2,2-diphenyl-1-picrylhydrazyl (DPPH); 2,2′-azino-bis-3-ethylbenzothiazoline-6-sulfonic acid (ABTS) assays; ferric reducing antioxidant power (FRAP) assays; and cupric reducing antioxidant capacity (CUPRAC) assays; 0.025–1 mg/mL for xanthine oxidase (XO) inhibitory activity (superoxide radical scavenging activity) assay). Stock sample test solutions filtered through 0.2 µm GHP polypropylene membrane filters (Pall Corporation, Ann Arbor, MI, USA) and diluted with methanol (0.25–2.5 mg/mL) were used for UPLC analyses.

### 2.5. Isolation and Structure Elucidation of Marker Compounds from the Bee Pollen Sample

**Figure 1:**
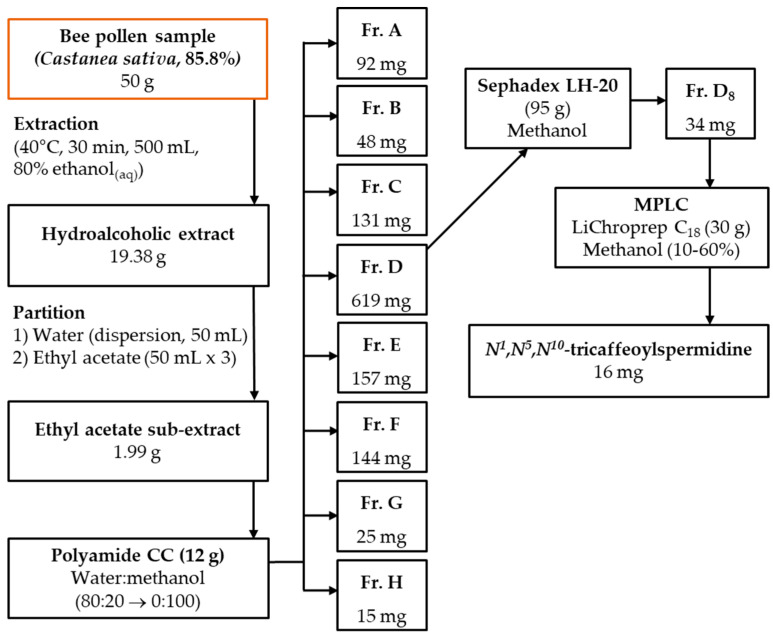
The isolation scheme of the targeted compound (*N*^1^,*N*^5^,*N*^10^-tricaffeoylspermidine).

The bee pollen sample (C1; 50 g) was extracted with 80% ethanol_(aq)_ (500 mL) for 30 min in an ultrasonic bath. After filtration, the solvent was evaporated under vacuum at 40 °C to yield hydroalcoholic extract (19.38 g), which was suspended in water (50 mL) and partitioned with ethyl acetate (50 mL × 3). The ethyl acetate fraction (1.99 g) was applied to polyamide column chromatography (CC) (12 g) and eluted with a water–methanol mixture (20–100% methanol) to give 8 main fractions (Fr. A–H; Figure 1). Fraction D (619 mg) was applied to the Sephadex LH-20 (95 g) CC with 600 mL of methanol to yield 8 fractions (Fr. D_1–8_). The targeted compound was purified from Fr. D_8_ (34 mg) by medium pressure liquid chromatography (MPLC, BÜCHI Labortechnik, Flawil, Switzerland) using a C_18_ MPLC column (30 g) and methanol–water (10–60% methanol) (Figure 1). The flow rate was 8 mL/min, and the run time was approximately 4 h. All isolation steps were performed at room temperature. The structure of the compound was elucidated by NMR (1D) and HRMS methods.

### 2.6. HPTLC Analyses

HPTLC analyses were performed on 20 cm × 10 cm glass-backed HPTLC silica gel 60 NH_2_ F_254S_ plates (Merck, Art. No. 1.13192). Solutions were applied to the plate as 8 mm bands, 8 mm from the bottom of the plate and 15 mm from the left edge, with a semi-automatic applicator Linomat 5 (Camag, Muttenz, Switzerland) equipped with a 100 µL Hamilton syringe. Three different sets of solutions were applied to the plates: (1) *N*^1^,*N*^5^,*N*^10^-tricaffeoylspermidine (200 µg/mL, 2 µL) and stock sample test solutions of bee pollen (20 mg/mL, 5 µL); (2) standard solutions (200 µg/mL, 2 µL: rutin, isoquercitrin, hyperoside, myricitrin, quercitrin, quercetin, and *N*^1^,*N*^5^,*N*^10^-tricaffeoylspermidine) and stock sample test solutions of androecia (20 mg/mL, 5 µL); (3) MIX (28.5 µg/mL, 8 µL) and stock sample test solutions of bee pollen and androecia samples (20 mg/mL, 5 µL). The plate was developed up to 8 cm in a saturated (20 min) twin-trough chamber (20 cm × 10 cm, Camag) with a developing solvent system containing ethyl acetate–formic acid–water (35:4:4, *v*/*v*/*v*).

The same derivatization procedure was used for analyses of all three sets of solutions. After drying the plate in a stream of cold air, the plate was heated on a TLC plate heater (Camag) at 100 °C for 3 min and immersed in an NP derivatization reagent, and after drying, it was also immersed in PEG reagent [24] by a Chromatogram Immersion Device III (Camag) for 3 s. An additional anisaldehyde–sulfuric acid derivatization reagent was used only for analyses of the 3rd set of solutions. In this case, the developed and dried plate was dipped for 2 s into anisaldehyde detection reagent by means of a Chromatogram Immersion Device III. Anisaldehyde (ANIS) detection reagent was prepared by mixing glacial acetic acid (20 mL) and methanol (170 mL). During cooling with cold water, 10 mL of sulfuric acid was added in a dropwise manner, and, subsequently, anisaldehyde (1 mL) was added to the mixture [25].

Documentation of the plate images was performed using a Visualiser (Camag) and the following illumination conditions: (1) 254 nm—before derivatization; (2) 366 nm—before derivatization and after derivatization with anisaldehyde or NP reagents, as well as after enhancement of the fluorescence with PEG; (3) white light (transmission mode)—after derivatization with anisaldehyde and after application of PEG reagent. The winCATS program was used to operate all the instruments (Camag, Version 128 1.4.8.2031).

### 2.7. UPLC Analysis

UPLC analysis was performed using the Nexera UHPLC series (Shimadzu, Kyoto, Japan), consisting of a quaternary pump, an autosampler, a thermostatted column compartment, and a photodiode array (PDA) detector. The UPLC system was operated by LabSolutions software (version 5.111). Analyses were carried out on a Poroshell 120 EC-C_18_ column (3 mm × 150 mm I.D., 2.7 µm particle size, Agilent (USA)). The column temperature was set to 25 °C. Mobile phase A [*o*-phosphoric acid–water (0.1:99.9, *v*/*v*)] and mobile phase B (acetonitrile) were degassed and filtered through 0.2 µm filter before analyses. The following gradient elution with different flow rates was applied: 17–17.2% B (0–2 min, 0.5 mL/min), 17.2–17.3% B (2–10 min, 0.5 mL/min), 17.3–20% B (10–11 min, 0.5 mL/min), 20–28% B (11–15 min, 0.5 mL/min), 28–40% B (15–25 min, 0.275 mL/min), 40–70% B (25–26 min, 0.2 mL/min), 70% B (26–27 min, 0.5 mL/min), and 70–17% B (27–28 min, 0.5 mL/min). The injection volume was 2 µL. Three acquisition wavelengths were used for quantitative analyses: 256 nm for rutin, hyperoside, isoquercitrin, quercitrin, and quercetin; 260 nm for myricitrin; 320 nm for *N*^1^,*N*^5^,*N*^10^-tricaffeoylspermidine. This newly developed UPLC method was validated according to the International Conference on Harmonisation (ICH) 1995 guidelines [26] and was used for the quantification of rutin, hyperoside, isoquercitrin, quercitrin, quercetin, myricitrin, and *N*^1^,*N*^5^,*N*^10^-tricaffeoylspermidine in androecia and bee pollen sample test solutions.

### 2.8. In Vitro Bioactivity Analyses

*In vitro* antioxidant activity (DPPH, FRAP, CUPRAC, and ABTS) and xanthine oxidase (XO) inhibitory (superoxide radical scavenging) activity were analyzed for bee pollen and androecia sample test solutions as well as for the isolated *N*^1^,*N*^5^,*N*^10^-tricaffeoylspermidine. The concentration ranges of bee pollen and androecia sample test solutions used for antioxidant activity assays and XO inhibitory activity assays were in the range 0.25–2.5 mg/mL and 0.025–1 mg/mL, respectively. The concentrations of *N*^1^,*N*^5^,*N*^10^-tricaffeoylspermidine were 50 µg/mL for antioxidant activity assays and 10–400 µg/mL for XO inhibitory activity assays. The antioxidant activities obtained were presented as mg of trolox equivalents (TE) per g of hydroalcoholic extract (mg TE/g extract) for samples and mg TE/g for *N*^1^,*N*^5^,*N*^10^-tricaffeoylspermidine. Half-maximal inhibitory concentration values (IC_50_) were calculated for XO inhibitory activity. Inhibition values (%) were calculated based on absorbances (A) measured against blank solutions by using the following formula:

Inhibition (%): (1 − (A_Sample_ − A_Sample Blank_)/(A_Control_ − A_Control Blank_)) × 100 (1)

The blanks, controls (substrate and enzyme solutions), and control blank (substrate solution) are described in detail in the following subsections.

#### 2.8.1. DPPH• Assay

After adding equal volumes (20 µL) of the diluted sample test solutions, trolox standard solutions (3.125–100 µg/mL), and methanol (as a blank) separately into wells of a 96-well microplate, DPPH solution (0.1 mM, 280 µL) was added, respectively, into each well. Incubation in the dark at room temperature for 30 min was followed by reading the absorbance at 530 nm [10].

#### 2.8.2. FRAP Assay

Equal volumes (20 µL) of diluted sample test solutions, trolox standard solutions (3.125–100 µg/mL), and water (blank) were added to separate wells of a 96-well microplate. A freshly prepared FRAP solution (280 µL of a mixture of iron (III) chloride solution (2 × 10^−2^ M), TPTZ solution (1 × 10^−2^ M) and sodium acetate buffer (pH 3.6) solution, 1:1:10, *v*/*v*/*v*) was added into each well. The absorbance was read at 593 nm after 6 min [27].

#### 2.8.3. CUPRAC Assay

Copper (II) sulfate pentahydrate (10 mM, 85 µL), neocuproine (7.5 mM, 85 µL), ammonium acetate buffer solution (85 µL, pH 7), and water (51 µL) were added into separate wells of a 96-well microplate. This was followed by the addition of either diluted sample test solutions (43 µL) or trolox standard solutions (3.125–200 µg/mL) or water (blank) into each well. After incubation at room temperature for 30 min, the absorbance was read at 450 nm [28].

#### 2.8.4. ABTS Assay

As in our previous study [10], a slightly modified ABTS assay [29] was applied. Before adding ABTS reagent (280 µL) into each well of a 96-well microplate plate, equal volumes (20 µL) of the diluted sample test solutions, trolox standard solutions (6.25–100 µg/mL), or methanol (blank) were added. After incubation at room temperature for 6 min, the absorbance was read at 734 nm.

#### 2.8.5. Xanthine Oxidase Inhibitory Activity

Equal volumes (20 µL) of diluted sample test solutions and allopurinol (10–200 µg/mL, used as a positive control) solution were placed into separate wells of a 96-well microplate. Addition of the substrate solution (a mixture of 0.4 mM xanthine and 0.24 mM NBT, 80 µL) was followed by the addition of XO solution (50 mU/mL in sodium phosphate buffer (pH 7.5), 80 µL). Hydrochloric acid (0.6 M, 80 µL) was used to terminate the reaction after 20 min of incubation at 37 °C. The absorbance was measured at 560 nm against a blank solution containing all reagents except XO [30].

### 2.9. HPTLC-Effect-Directed Analyses (EDAs)

#### 2.9.1. HPTLC-DPPH•

HPTLC-DPPH• assays were performed on two HPTLC NH_2_ F_254S_ plates. One of the following two sets of solutions was applied to either of the plates: (1) *N*^1^,*N*^5^,*N*^10^-tricaffeoylspermidine (200 µg/mL, 2 µL) and sample test solutions of bee pollen (20 mg/mL, 5 µL); (2) standard solutions (200 µg/mL, 2 µL: rutin, isoquercitrin, hyperoside, myricitrin, quercitrin, quercetin, and *N*^1^,*N*^5^,*N*^10^-tricaffeoylspermidine) and androecia sample test solutions (20 mg/mL, 5 µL). Other application conditions as well as developing and drying (after development) conditions were the same as those described in Section 2.6. HPTLC analyses. After development and drying, the plates were dipped into DPPH solution (0.1% methanolic solution) for 3 s using a Chromatogram Immersion Device III (Camag). After drying in the air in the dark for 30 min, the plates were documented immediately (t = 0 min) and after 5, 10, 15, 20, 30, 45, 60, 90, and 120 min under white light in transmission mode using a Visualiser (Camag). Between intervals, the plates were stored in the dark. Antioxidant compounds were detected as yellow-colored bands on the purple background.

#### 2.9.2. HPTLC-Xanthine Oxidase Inhibitory Activity

HPTLC-XO inhibitory activity was performed on two silica gel NH_2_ F_254S_ plates. One of the two sets of solutions was applied to separate plates: (1) *N*^1^,*N*^5^,*N*^10^-tricaffeoylspermidine (1 mg/mL, 5 µL) and sample test solutions of bee pollen (50 mg/mL, 5 µL); (2) standard solutions (1 mg/mL, 5 µL: rutin, isoquercitrin, hyperoside, myricitrin, quercitrin, quercetin, and *N*^1^,*N*^5^,*N*^10^-tricaffeoylspermidine) and androecia sample test solutions (50 mg/mL, 5 µL). Other application conditions, as well as developing and drying (after development) conditions, are described in Section 2.6. HPTLC analyses. After development, allopurinol (1 mg/mL, 15 µL), which was used as the positive control, was applied to the HPTLC plates. The plates were then dipped into a derivatization chamber containing a mixture of phosphate buffer solution (pH 7.6), EDTA (1 mM), NBT (1 mM), and XO (0.1 U/mL) using a Chromatogram Immersion Device III (Camag). After incubating at 37 °C for 30 min in the dark and drying in the air, the plates were then immersed into a phosphate buffer solution containing xanthine (1.5 mM) and incubated for 30 min at 37 °C in the dark. The plates were documented immediately (t = 0 min) and after 5, 10, 15, 20, 30, 45, 60, 90, and 120 min under white light in transmission mode using a Visualiser (Camag). Between intervals, the plates were stored in the dark. The compounds having XO inhibitory activity were then detected as white/yellow zones on a purple background [10].

### 2.10. Image Analyses

Images of the HPTLC plates documented as described in Section 2.6 HPTLC analyses and Section 2.9. HPTLC-Effect-Directed Analyses (EDAs) were converted to a different format using winCATS software (Camag, Version 1.4.9.2001) and transformed to videodensitograms in absorption mode or fluorescence mode using VideoScan TLC/HPTLC Evaluation Software (Version 1.02.00) (Camag). Absorption mode was applied only in the case of the image of the plate documented at white light after derivatization with anisaldehyde reagent. Fluorescence mode was applied in all other cases where the images of the plates were documented: (1) at 366 nm after derivatization with anisaldehyde reagent, as well as after enhancement of the fluorescence with PEG that followed derivatization with NP reagent; (2) at white light after HPTLC-DPPH analyses. Videodensitograms obtained after applying NP/PEG or anisaldehyde reagents were used to generate the profile comparison for comparing chromatographic fingerprint profiles of sample test solutions prepared from bee pollen and androecia samples. Additionally, videodensitograms obtained after HPTLC-DPPH analyses were also used for the integration and calculation of peak areas. The total peak areas obtained for each of the tracks were used for a comparison of the analyzed androecia and bee pollen samples and standard compounds.

### 2.11. Statistical Analyses

Assays for bioactivity and quantitative analyses were performed in triplicates. Microsoft Excel 2013 was used to calculate the average values of the replicates and standard deviations (SD), stated as the average value ± SD. One-way analysis of variance (ANOVA) with Tukey’s test, performed by Minitab 17, was applied to evaluate statistically significant differences between the means of bioactivity test results (*p* < 0.05).

## 3. Results and Discussion

### 3.1. Isolation and Structural Elucidation of N^1^,N^5^,N^10^-Tricaffeoylspermidine from Chestnut Bee Pollen

Polyamines (putrescine, spermidine, and spermine) and their derivatives conjugated to hydroxycinnamic acids were found in some bee pollen samples [2,3,4,5,6,31,32,33]. After isolation of *N*^1^,*N*^5^,*N*^10^-tricaffeoylspermidine from chestnut bee pollen, its structure was elucidated according to the HRMS (Figure S1), ^13^C, and ^1^H NMR data (Table 2; Figures S2 and S3). In this study, *N*^1^,*N*^5^,*N*^10^-tricaffeoylspermidine was found as a common compound in bee pollen and androecia samples, and it was found for the first time in bee pollen samples originating from *Salix* spp. In previous studies, *N*^1^,*N*^5^,*N*^10^-tricaffeoylspermidine was identified in bee pollen originating from *Castanea sativa*, *Quercus mongolica*, *Phellodendron chinense*, *Fagopyrum esculentum*, *Prunus armeniaca*, *Camellia sinensis*, and *Cocos nucifera* [5,34,35]. *N*^1^,*N*^5^,*N*^10^-tricaffeoylspermidine was found in male but not in female *Salix purpurea* catkins [36]. *N*^1^,*N*^5^,*N*^10^-tricaffeoylspermidine was identified in the pollen of *Quercus* species such as *Q. rubra*, *Q. coccinea*, *Q. dentata*, *Q. cerris*, *Q. macrocarpa*, *Q. palustris*, *Q. velutina*, *Q. bicolor*, *Q. prinus*, *Q. robur*, and *Q. phellos* [19,37]. To the best of our knowledge, this is the first report of the identification of *N*^1^,*N*^5^,*N*^10^-tricaffeoylspermidine in *C. sativa*, *S. alba*, and *Q. pubescens* androecia.

### 3.2. HPTLC Chemical Profiling

A new HPTLC method was developed and applied for evaluating chemical profiles of bee pollen samples (with the dominant pollen of *C. sativa*, *Salix* spp., and *Quercus* spp.) and from androecia (*C. sativa*, *S. alba*, and *Q. pubescens*) samples. The fingerprints were investigated on HPTLC NH_2_ F_254S_ plates before (at 254 nm and at 366 nm) and after derivatization with NP detection reagent (at 366 nm), followed by enhancement and stabilization of fluorescence with PEG detection reagent (at 366 nm and at white light), and after derivatization with anisaldehyde reagent (at 366 nm and at white light). Use of the amino plates provided a better chromatographic separation, in particular of the spermidine derivatives. *N*^1^,*N*^5^,*N*^10^-tricaffeoylspermidine was well separated from other spermidine derivatives on the HPTLC amino plate but not on the HPTLC silica gel plate [33].

#### 3.2.1. HPTLC Chemical Profiling of Bee Pollen Samples

**Figure 2:**
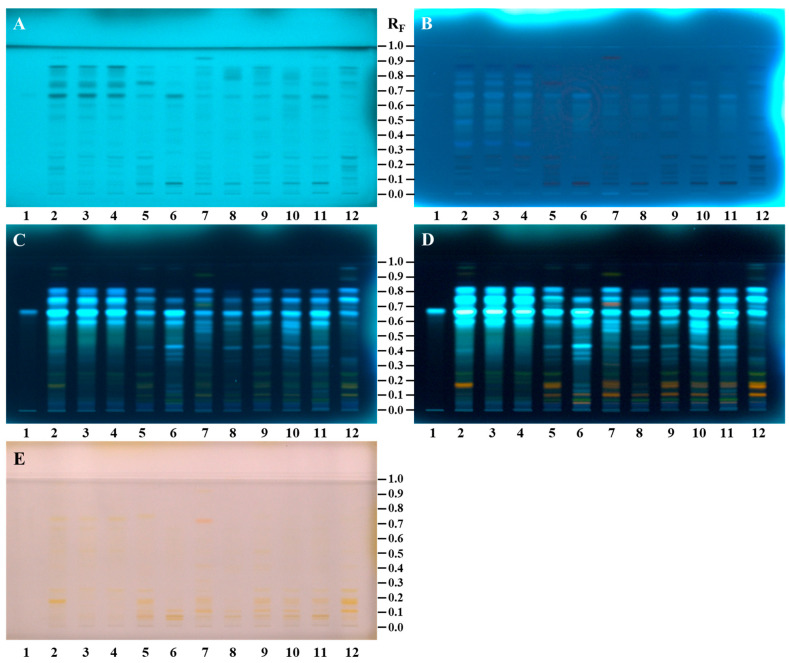
HPTLC NH_2_ F_254S_ plate developed with ethyl acetate–formic acid–water (35:4:4, *v*/*v*/*v*), documented before (at 254 nm (**A**) and 366 nm (**B**)) and after derivatization with NP reagent (at 366 nm (**C**)), followed by enhancement and stabilization of the zones with PEG reagent (at 366 nm (**D**) and white light (**E**)). Applications per band: track 1: *N*^1^,*N*^5^,*N*^10^-tricaffeoylspermidine (0.4 μg); track 2–4: C-bee pollen (C1–C3, 0.1 mg); track 5–11: S-bee pollen (S1–S7, 0.1 mg); track 12: Q-bee pollen (Q1, 0.1 mg).

Differences in the chemical fingerprints of the bee pollen samples (20 mg/mL, 5 µL) of different botanical origin were first compared with *N*^1^,*N*^5^,*N*^10^-tricaffeoylspermidine (200 µg/mL, 2 µL) after the separation on the HPTLC NH_2_ F_254S_ plate (Figure 2). *N*^1^,*N*^5^,*N*^10^-tricaffeoylspermidine (isolated in this study) at R_F_ ≈ 0.67 was detected as a dark grey zone at 254 nm (Figure 2A, track 1) and a blue zone at 366 nm (Figure 2B, track 1) before derivatization. This R_F_ zone became much more intense after derivatization with NP reagent (light-blue fluorescent zone at 366 nm (Figure 2C, track 1)) and even more intense after the enhancement of fluorescence with PEG reagent (light-blue fluorescent zones at 366 nm (Figure 2D, track 1)) but was not visible at white light (Figure 2E, track 1). Zones at the same R_F_ with the same colors (Figure 2A–D, tracks 2–12), except at white light, detected in bee pollen samples indicated the presence of *N*^1^,*N*^5^,*N*^10^-tricaffeoylspermidine. Among bee pollen samples, the most intensive zones of *N*^1^,*N*^5^,*N*^10^-tricaffeoylspermidine were observed for all C-bee pollen (Figure 2, tracks 2–4).

After development, the same band pattern (R_F_ zones at ≈ 0.87, 0.85, 0.81, 0.76, 0.74, 0.67, 0.60, 0.53, 0.25, 0.22, 0.19, 0.14, and 0.06) was observed at 254 nm and 366 nm for all bee pollen samples (Figure 2A,B, tracks 2, 3, and 4) with *C. sativa* as the dominant pollen (from 85.8% to 98%) as confirmed by the palynological analyses (Table 1). After use of NP/PEG reagents, the same pattern with the most intense zones (R_F_ ≈ 0.60, 0.67, 0.74, and 0.81) was observed at 366 nm for all bee pollen samples from different geographical regions and even countries (Figure 2D, track 2—İzmit (Türkiye), track 3—Artvin (Türkiye), and track 4—Ptuj (Slovenia)). Additionally, before derivatization, blue zones (R_F_ ≈ 0.35) were observed at 366 nm only for C-bee pollen (Figure 2B, tracks 2, 3, and 4).

In addition to the light-blue fluorescent zone of *N*^1^,*N*^5^,*N*^10^-tricaffeoylspermidine (R_F_ ≈ 0.67), the pattern of light-blue fluorescent zones was observed at R_F_ ≈ 0.60, 0.74, and 0.81 in all bee pollen samples at 366 nm after derivatization with NP reagent (Figure 2C, tracks 2–12). However, the colors of the zones at R_F_ ≈ 0.81 in the tracks of two *Salix* spp. samples (Figure 2C,D, tracks 6 and 8) were a shade darker compared to the colors of the zones at the same R_F_ in the tracks of the other nine samples (Figure 2C,D, tracks 2–5, 7, and 9–12). All these zones (R_F_ ≈ 0.60, 0.74, and 0.81) became much more intense at 366 nm after the application of PEG reagent. These zones, together with the zone of *N*^1^,*N*^5^,*N*^10^-tricaffeoylspermidine (R_F_ ≈ 0.67), were found to be more intense in the C-bee pollen compared to other bee pollen samples (Figure 2D). Only C-bee pollen showed the pattern of three zones at R_F_ values ≈ 0.19, 0.22, and 0.25 at 254 nm (dark grey zones, Figure 2A, tracks 2–4) and 366 nm (black zones, Figure 2B, tracks 2–4) after development and at 366 nm after using NP (green zones, Figure 2C, tracks 2–4) and PEG reagents (green zones, Figure 2D, tracks 2–4). At the same conditions (366 nm, NP/PEG reagents), the green zone at R_F_ ≈ 0.22 was detected also in Q-bee pollen (Figure 2D, track 12) but not in S-bee pollen (Figure 2D, tracks 5–11). Additionally, the green zones at R_F_ ≈ 0.25 (Figure 2C,D) were observed at 366 nm in all bee pollen samples except S2 (Figure 2C,D, track 6) and S4 (Figure 2C,D, track 8). Orange zones were detected at R_F_ ≈ 0.25 with white light in S-bee pollen (Figure 2E, tracks 5–11) and Q-bee pollen (Figure 2E, track 12) but not in C-bee pollen (Figure 2E, tracks 2–4). Green zones at R_F_ ≈ 0.07 were detected at 366 nm after application of PEG reagent in all tracks of *Salix* spp. (Figure 2D, tracks 5–11) and *C. sativa* (Figure 2D, tracks 2–4) but were not detected in *Quercus* spp. (Figure 2D, track 12). At the same R_F_ (≈0.07), brown zones were observed at white light after application of PEG reagent in S-bee pollen (Figure 2E, tracks 5–11) but were not detected in C-bee pollen (Figure 2E, tracks 2–4) and Q-bee pollen (Figure 2E, track 12).

After derivatization with NP reagent, a pattern of three light-blue fluorescent zones at R_F_ values ≈ 0.35, 0.38, and 0.43 (Figure 2C, tracks 5–11) was observed at 366 nm only for S-bee pollen (Figure 2C, tracks 5–11). The intensity of these zones further increased after the application of PEG reagent (Figure 2D, tracks 5–11). Additionally, one green- (R_F_ ≈ 0.92), one red- (R_F_ ≈ 0.72), and two orange-colored (R_F_ ≈ 0.41 and 0.31) zones were detected at 366 nm after using PEG reagent in only one (S3; Figure 2D, track 7) of the seven S-bee pollen samples (Figure 2D, tracks 5–11). These four zones were detected as orange with white light after application of PEG reagent (Figure 2E, track 7). Zones at R_F_ ≈ 0.11, colored yellow after using NP reagent and orange after using PEG reagent, were detected at 366 nm in S-bee pollen (Figure 2D, tracks 5–11); *C. sativa* (Figure 2D, track 2); and *Quercus* spp. (Figure 2D, track 12). These zones were also orange at white light after the application of PEG reagent in all bee pollen samples from *Salix* spp. (Figure 2E, tracks 5–11) and *Quercus* spp. (Figure 2E, track 12) but not in C-bee pollen (Figure 2E, tracks 2–4). Zones at R_F_ ≈ 0.16 and 0.19, colored yellow after derivatization with NP reagent and orange after application of PEG reagent, were detected at 366 nm in S-bee pollen (Figure 2C,D, tracks 5, 7, 9–11), C-bee pollen (Figure 2C,D, track 2), and Q-bee pollen (Figure 2C,D, track 12). These zones were also orange at white light after the application of PEG reagent (Figure 2E, tracks 2, 5, 7, and 9–12). After the application of PEG reagent, orange-brown zones (R_F_ ≈ 0.05; Figure 2E, tracks 5–11)) and yellow zones (R_F_ ≈ 0.74, 0.52; Figure 2E, tracks 2–4) were detected with white light in S-bee pollen and C-bee pollen, respectively.

In general, bee pollen samples originating from the same botanical source as *C. sativa* (Figure 2, tracks 2–4) and *Salix* spp. (Figure 2, tracks 5–11) showed similar HPTLC profiles. However, due to different percentages of the dominant botanical source pollen (85.8–98.0% for C-bee pollen and 45.1–89.8% for S-bee pollen), HPTLC profiles of bee pollen from the same botanical source showed different additional zones (Figure 2) related to pollen of other botanical sources present in the samples.

#### 3.2.2. HPTLC Chemical Profiling of Androecia Samples

**Figure 3:**
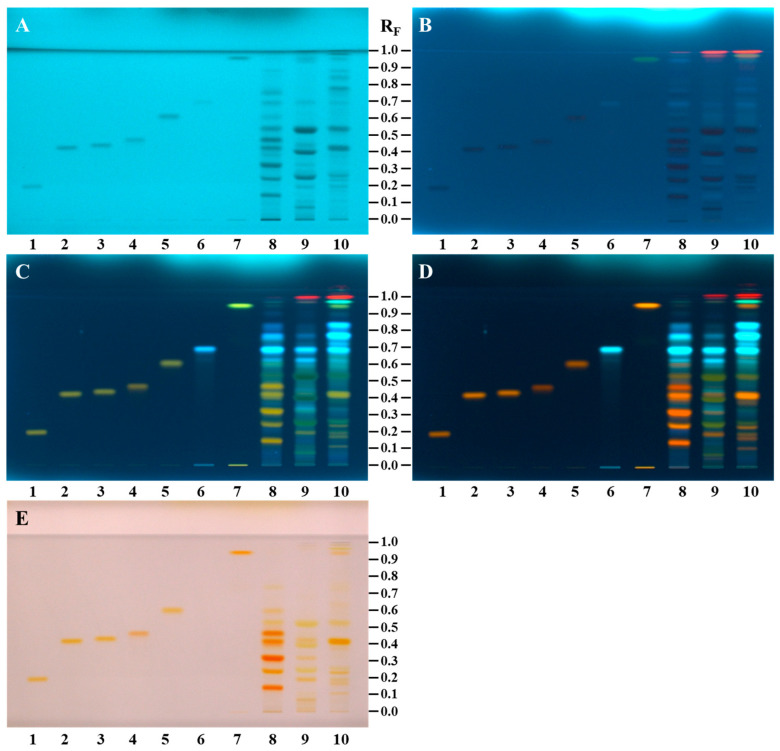
HPTLC NH_2_ F_254S_ plate developed with ethyl acetate–formic acid–water (35:4:4, *v*/*v*/*v*), documented before (at 254 nm (**A**) and 366 nm (**B**)) and after derivatization with NP reagent (**C**), followed by enhancement and stabilization of the zones with PEG reagent (at 366 nm (**D**) and white light (**E**)). Applications per band: track 1: rutin (0.4 μg); track 2: hyperoside (0.4 μg); track 3: isoquercitrin (0.4 μg); track 4: myricitrin (0.4 μg); track 5: quercitrin (0.4 μg); track 6: *N*^1^,*N*^5^,*N*^10^-tricaffeoylspermidine (0.4 μg); track 7: quercetin (0.4 μg); track 8: *C. sativa* androecia (0.1 mg); track 9: *S. alba* androecia (0.1 mg); track 10: *Q. pubescens* androecia (0.1 mg).

Differences in the chemical fingerprints of the androecia samples (20 mg/mL, 5 µL) of *C. sativa*, *S. alba*, and *Q. pubescens* were compared and evaluated with the seven compounds investigated (standards of rutin, hyperoside, isoquercitrin, myricitrin, quercitrin, quercetin, and *N*^1^,*N*^5^,*N*^10^-tricaffeoylspermidine) (200 µg/mL, 2 µL) after the separation on the HPTLC NH_2_ F_254S_ plate (Figure 3). In this way, the potential of these seven compounds as identification markers for distinguishing among the three floral sources was evaluated. After development, all seven compounds (rutin: R_F_ ≈ 0.19, track 1; hyperoside: R_F_ ≈ 0.42, track 2; isoquercitrin: R_F_ ≈ 0.43, track 3; myricitrin: R_F_ ≈ 0.47, track 4; quercitrin: R_F_ ≈ 0.60, track 5; *N*^1^,*N*^5^,*N*^10^-tricaffeoylspermidine: R_F_ ≈ 0.67, track 6; quercetin: R_F_ ≈ 0.93, track 7) were detected at 254 nm as grey zones (Figure 3A). Although the amounts (0.4 μg) of these compounds on the plate were the same, the zone of *N*^1^,*N*^5^,*N*^10^-tricaffeoylspermidine was less intense (Figure 3A, track 6) than the zones of the six flavonoids (Figure 3A, tracks 1–5, 7). At 366 nm (Figure 3B), the zones for five of these compounds (rutin (track 1), hyperoside (track 2), isoquercitrin (track 3), myricitrin (track 4), and quercitrin (track 5)) were dark blue, while the zones of *N*^1^,*N*^5^,*N*^10^-tricaffeoylspermidine (track 6) and quercetin (track 7) were light blue and light green, respectively. After derivatization with NP, six compounds (Figure 3C, tracks 1–5 and 7) were detected as yellow zones at 366 nm, while *N*^1^,*N*^5^,*N*^10^-tricaffeoylspermidine (Figure 3C, track 6) was detected as a light-blue fluorescent zone. Similarly, after application of PEG reagent, six compounds (Figure 3D, tracks 1–5 and 7) were detected as orange zones at 366 nm and at white light, but *N*^1^,*N*^5^,*N*^10^-tricaffeoylspermidine (Figure 3D, track 6) was detected as a light-blue fluorescent zone at 366 nm but was not detected with white light (Figure 3E, track 6).

The presence of an *N*^1^,*N*^5^,*N*^10^-tricaffeoylspermidine zone at R_F_ ≈ 0.67 was observed as one of the most intense zones for all androecia samples (Figure 3, tracks 8–10). The zone at the R_F_ ≈ 0.19 of rutin (Figure 3, track 1) was detected in androecia *S. alba* (Figure 3, track 9) and *Q. pubescens* (Figure 3, track 10) but not in *C. sativa*. The zone at the R_F_ ≈ 0.42 of hyperoside (Figure 3, track 2) was detected in *C. sativa* (Figure 3, track 8) and *Q. pubescens* (Figure 3, track 10) androecia. The zone at the R_F_ ≈ 0.43 of isoquercitrin (Figure 3, track 3) was detected in all androecia samples (*C. sativa* (Figure 3, track 8), *S. alba* (Figure 3, track 9), and *Q. pubescens* (Figure 3, track 10). Zones of myricitrin (R_F_ ≈ 0.47, Figure 3, track 4) and quercitrin (R_F_ ≈ 0.60) (Figure 3, track 5) were detected only in *C. sativa* androecia (Figure 3, track 8). The zone at R_F_ ≈ 0.93 of quercetin (Figure 3, track 7) was detected only in *Q. pubescens* androecia (Figure 3, track 10). Differently colored zones were detected at the same R_F_ (≈0.93) in *C. sativa* androecia (Figure 3, track 8) after development (Figure 3A: grey at 254 nm; Figure 3B: light blue at 366 nm) and after using NP reagent (Figure 3C: yellow at 366 nm) and PEG reagent (Figure 3D: orange at 366 nm). At the same R_F_ (≈0.93) the zone was detected also in *S. alba* androecia at 254 nm (grey zone, (Figure 3A, track 9)) and 366 nm (red zone, (Figure 3B, track 9)) but only after development. The zones at R_F_ ≈ 0.86 were detected in *C. sativa* and *Q. pubescens* androecia at 254 nm (grey, Figure 3A, tracks 8 and 10) and 366 nm (violet, Figure 3B, tracks 8 and 10) only after development. The zones at R_F_ ≈ 0.96 were detected after development at 254 nm only in *Q. pubescens* androecia (grey zone (Figure 3A, track 10)) but at 366 nm in *C. sativa* (red zone (Figure 3B, track 8)), *S. alba* (red zone (Figure 3B, track 9)), and *Q. pubescens* androecia (yellow zone (Figure 3B, track 10)). After using NP reagent, the zone at the same R_F_ (≈0.96) was detected at 366 nm in *C. sativa* (green-blue zone, Figure 3C, track 8), *S. alba* (orange zone, Figure 3C, track 9), and *Q. pubescens* androecia (green-blue zone, Figure 3C, track 10). The green-blue (after NP reagent (Figure 3C, tracks 8 and 10)) or blue (after PEG reagent (Figure 3D, tracks 8 and 10)) colored zones were detected at 366 nm in the *C. sativa* and *Q. pubescens* androecia. At 366 nm, the zone at the same R_F_ in the *S. alba* androecia (Figure 3C, track 9) was yellow-orange or yellow after using NP and PEG reagents (Figure 3D, track 9), respectively. The same R_F_ zone in *S. alba* and *Q. pubescens* androecia was yellow at white light after using PEG reagent. After development, zones at R_F_ ≈ 0.77 were detected in *C. sativa* (light blue at 366 nm, (Figure 3B, track 8)) and *Q. pubescens* androecia (grey at 254 nm (Figure 3A, track 10) and light blue at 366 nm (Figure 3B, track 10)). Several other zones were also detected in *C. sativa*, *S. alba*, and *Q. pubescens* androecia after development (at 254 and 366 nm) as well as after using NP (at 366 nm) and PEG reagents (at 366 nm and at white light). Apart from the light-blue fluorescent zone of *N*^1^,*N*^5^,*N*^10^-tricaffeoylspermidine (R_F_ ≈ 0.67), three additional light-blue fluorescent zones at R_F_ ≈ 0.60, 0.74, and 0.81 were detected at 366 nm after using NP and were common to all androecia samples (Figure 3C, tracks 8–10).

Additional blue fluorescent zones at R_F_ ≈ 0.43 and 0.35 (Figure 3D, track 9) were observed for *Salix alba* androecia at 366 nm after using NP/PEG reagents. Dark blue zones at R_F_ ≈ 0.35 and 0.21 (Figure 3C,D, track 8) were detected in *C. sativa* androecia at 366 nm after using NP reagent. These zones became more intense after using PEG reagent. The zones at R_F_ ≈ 0.25 were detected in *S. alba* and *Q. pubescens* androecia (Figure 3, tracks 9 and 10) after development (at 254 nm—Figure 3A: dark grey and grey, respectively; dark blue at 366 nm Figure 3B) and after using NP (green at 366 nm—Figure 3C) and PEG reagents (yellow at white light—Figure 3E and at 366 nm—Figure 3D).

The zones at R_F_ ≈ 0.24 were detected in *C. sativa* and *Q. pubescens* androecia (Figure 3, tracks 8 and 10) as dark grey at 254 nm (Figure 3A) and dark blue at 366 nm (Figure 3B) after development, as orange with white light (Figure 3E), and at 366 nm (Figure 3D) after using PEG reagent. The zone at R_F_ ≈ 0.16 detected only in *Q. pubescens* androecia (Figure 3, track 10) was grey at 254 nm (Figure 3A) and 366 nm (Figure 3B) after development, yellow at 366 nm after using NP reagent (Figure 3C), and orange at 366 nm (Figure 3D) and with white light (Figure 3E) after using PEG reagent. Light-blue zones (R_F_ ≈ 0.15 and 0.35) detected at 366 nm in *S. alba* androecia (Figure 3B, track 9) after development became more intense after using NP (Figure 3C) and PEG reagents (Figure 3D). The zone at R_F_ ≈ 0.15 that was also observed in *C. sativa* androecia (Figure 3, track 8) was dark grey at 254 nm (Figure 3A) and dark blue at 366 nm (Figure 3B) after development and orange at 366 nm (Figure 3D) and at white light (Figure 3E) after using PEG reagent.

Zones at R_F_ ≈ 0.32 were detected only in *C. sativa* and *S. alba* androecia (Figure 3, tracks 8 and 9) after development (grey at 254 nm (Figure 3A) and dark blue at 366 nm (Figure 3B)) and after derivatization with NP reagent (orange at 366 nm (Figure 3C)). The application of PEG reagent resulted in more intense orange zones at 366 nm (Figure 3D), which were detected as orange with white light for *C. sativa* (Figure 3E, track 8) or yellow for *S. alba* (Figure 3E, track 9) androecia. The zone at R_F_ ≈ 0.38 of the track of *C. sativa* androecia (Figure 3, track 8) was detected only after development (grey zone at 254 nm (Figure 3A) and dark grey zone at 366 nm (Figure 3B)) and after using NP reagent (yellow zone at 366 nm (Figure 3C)), but it was not detected at 366 nm after using PEG reagent because of the interference of another highly intensive zone. An additional zone at R_F_ ≈ 0.37 was observed for *Q. pubescens* androecia (Figure 3, track 10) at 366 nm (orange zone, Figure 3D) and at white light (yellow zone, Figure 3E) after using PEG reagent.

Zones at R_F_ ≈ 0.39 and 0.52 were found in *S. alba* androecia (Figure 3, track 9) at 254 nm (dark grey, Figure 3A) and 366 nm (dark blue, Figure 3B) after development and after using NP (black-green at 366 nm Figure 3C) and PEG (green at 366 nm (Figure 3D) and yellow at white light Figure 3E) reagents. The zones at R_F_ ≈ 0.53 were detected in *C. sativa* and *Q. pubescens* androecia after development (dark grey at 254 nm (Figure 3A, tracks 8 and 10) and dark blue at 366 nm (Figure 3B, tracks 8 and 10)) and after derivatization with NP reagent (yellow at 366 nm (Figure 3C, tracks 8 and 10)). After using PEG reagent, yellow zones (R_F_ ≈ 0.53) were detected at white light for *C. sativa* and *Q. pubescens* androecia (Figure 3E, tracks 8 and 10) and at 366 nm for *Q. pubescens* androecia (Figure 3D, track 10), but orange zones were detected at 366 nm at the same R_F_ value for *C. sativa* androecia (Figure 3D, track 8). After derivatization with NP reagent, light-blue zones at R_F_ ≈ 0.58 and 0.71 were detected at 366 nm in *S. alba* (Figure 3C, track 9) and *Q. pubescens* androecia (Figure 3C, track 10), and using PEG reagent resulted in enhancement of the light-blue fluorescence of these zones. Zones at R_F_ ≈ 0.11 were detected in *S. alba* and *Q. pubescens* androecia (Figure 3, tracks 9 and 10) after development (grey at 254 nm (Figure 3A) and dark blue at 366 nm (Figure 3B)). After using NP reagent, the yellow zones (R_F_ ≈ 0.11) were observed at 366 nm for *S. alba* and *Q. pubescens* androecia (Figure 3C, tracks 9 and 10). After using PEG reagent, this R_F_ zone was observed for *S. alba* and *Q. pubescens* androecia as yellow and orange at 366 nm (Figure 3D, track 9) and at white light (Figure 3E, track 10), respectively. The zone at R_F_ ≈ 0.07 was observed only in androecia of *S. alba* (Figure 3, track 9) after development (dark grey at 254 nm (Figure 3A) and dark blue at 366 nm (Figure 3B)) and after using NP (yellow at 366 nm (Figure 3C)) and PEG reagents (green at 366 nm (Figure 3D) and brown orange at white light (Figure 3E)). This is the first study comparing the chemical composition of androecia belonging to apetalous trees including *C. sativa*, *S. alba*, and *Q. pubescens*.

#### 3.2.3. Comparisons of HPTLC Fingerprints of Bee Pollen and Androecia of *C. sativa*, *S. alba*, and *Q. pubescens*

HPTLC is a powerful analytical technique enabling the detection of various compounds at the same time using different derivatization reagents. In general, NP/PEG reagents are used for detecting phenolic compounds whereas an anisaldehyde reagent is not so specific but it is a more universal reagent used for detecting terpenic compounds and phenolic compounds as catechins [25,38].

**Figure 4:**
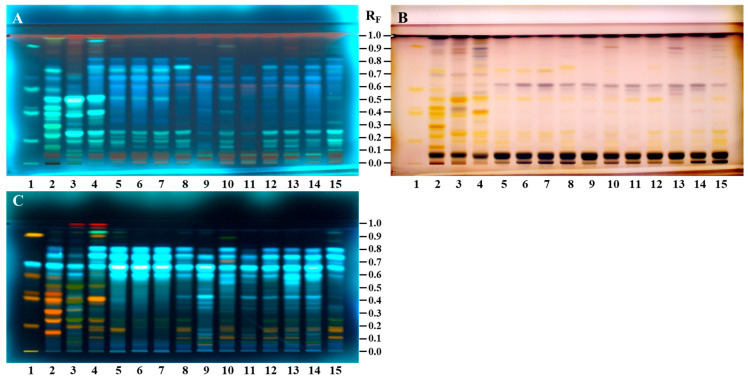
HPTLC NH_2_ F_254S_ plate developed with ethyl acetate–formic acid–water (35:4:4, *v*/*v*/*v*), documented after derivatization with anisaldehyde/H_2_SO_4_ reagent (at 366 nm (**A**) and white light (**B**)) and after derivatization with NP/PEG reagents (at 366 nm (**C**)). Applications per band: track 1: Standard MIX; track 2: *C. sativa* androecia (0.1 mg); track 3: *S. alba* androecia (0.1 mg); track 4: *Q. pubescens* androecia (0.1 mg); tracks 5–7: C-bee pollen (C1–C3, 0.1 mg); tracks 8–14: S-bee pollen (S1–S7, 0.1 mg); track 15: Q-bee pollen (Q1, 0.1 mg).

Bee pollen is a processed product of flower grains. Even if processing causes some differences in the morphological and chemical properties of the flower pollen, there is a strong relationship between bee pollen and its botanical source in terms of chemical composition. HPTLC fingerprints of bee pollen and *C. sativa*, *S. alba*, and *Q. pubescens* androecia were compared under 366 nm after application of NP/PEG and anisaldehyde (Figure 4).

All fingerprints were also analyzed under white light after post-chromatographic derivatization with an anisaldehyde reagent. Detailed information for the compounds found in bee pollen and androecia samples before and after using NP/PEG reagent is given in Section 3.2.1. HPTLC Chemical Profiling of Bee Pollen Samples and Section 3.2.2. HPTLC Chemical Profiling of Androecia Samples. This section focuses mainly on the chromatographic zones of compounds important for bee pollen of the same botanical origin and identification of their botanical sources. This is the first study to evaluate chemical composition differences between *C. sativa* (Türkiye and Slovenia), *Salix* spp. (Türkiye and Slovenia), and *Quercus* spp. (Türkiye) bee pollen samples and *C. sativa*, *S. alba*, and *Q. pubescens* androecia to determine the botanical origin of bee pollen.

HPTLC fingerprints of bee pollen and *C. sativa*, *S. alba*, and *Q. pubescens* androecia obtained after application of NP/PEG (at 366 nm) and anisaldehyde (ANIS, at 366 nm and white light) are given in Figure 4. Additionally, the common chromatographic zones found in the bee pollen samples and related botanical sources (Figure 4) are summarized in Table 3. For all bee pollen and androecia samples, three common light-blue zones (at R_F_ ≈ 0.60, 0.67 (*N*^1^,*N*^5^,*N*^10^-tricaffeoylspermidine), and 0.74) were detected at 366 nm after using NP/PEG reagents (Figure 4C, Table 3). Using anisaldehyde reagent, two additional common zones (at R_F_ ≈ 0.07 black-brown at 366 nm (Figure 4A, Table 3) and brown under white light (Figure 4B, Table 3) and at R_F_ ≈ 0.61 violet color zone only under white light (Figure 4B, Table 3)) were detected in all bee pollen and androecia samples. Apart from common characteristic zones for all bee pollen and androecia samples, anisaldehyde derivatization enabled the detection of compounds that could be used as markers for identification. Accordingly, the following two zones were found only Q-bee pollen and *Q. pubescens* androecia: green-blue zone at R_F_ ≈ 0.93 (at 366 nm, Figure 4A, Table 3) and violet at R_F_ ≈ 0.91 (under white light, Figure 4B, Table 3). The following three characteristic zones were detected in S-bee pollen and *S. alba* androecia using NP/PEG at 366 nm (Figure 4C, Table 3): green zone at R_F_ ≈ 0.07 and light-blue zones at R_F_ ≈ 0.35 and 0.43.

**Figure 5:**
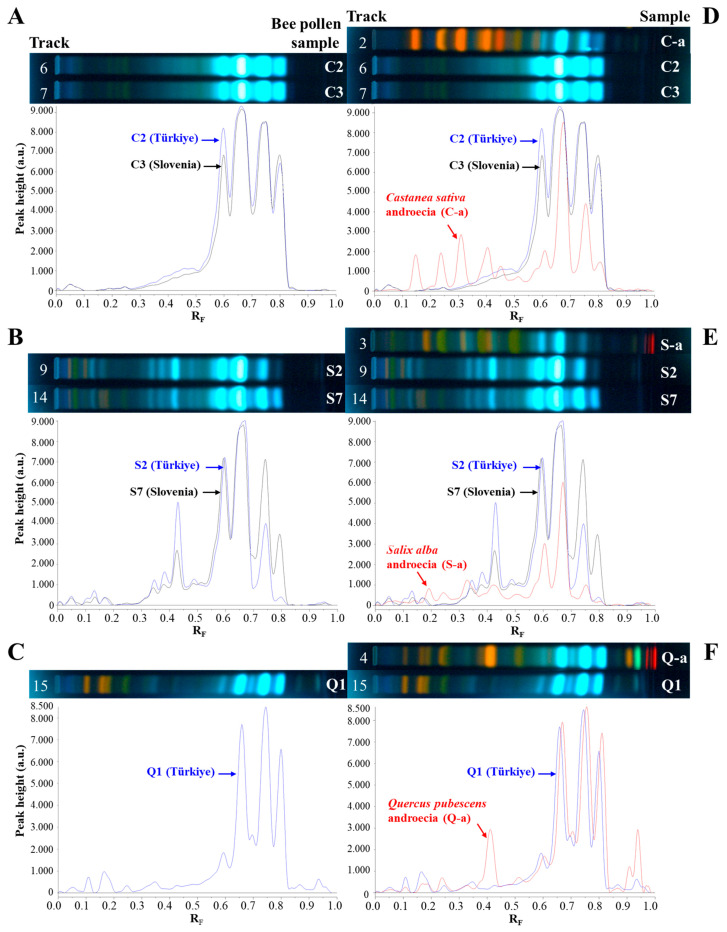
HPTLC fingerprints based on chromatograms at 366 nm after NP/PEG and videodensitogram profiles obtained from chromatograms (Figure 4) by image analysis in fluorescence mode. (**A**) C-bee pollen (C2 and C3), (**B**) S-bee pollen (S2 and S7), (**C**) Q-bee pollen (Q1), (**D**) C2 and C3 bee pollen and *C. sativa* androecia (C-a), (**E**) S2 and S7 bee pollen and *S. alba* androecia (S-a), (**F**) Q1 bee pollen and *Q. pubescens* androecia (Q-a). Application amounts of all samples per band: 0.1 mg.

Comparisons were performed using HPTLC fingerprints based on chromatograms and videodensitogram profiles obtained from chromatograms by image analysis in fluorescence mode. Bee pollen samples from Türkiye and Slovenia having the same botanical origin with the highest percentage of dominant pollen grains (Table 1) were compared, and their profiles obtained at 366 nm after using NP/PEG were compared with *C. sativa*, *S. alba*, and *Q. pubescens* androecia profiles obtained under the same conditions (Figure 5). The profiles of C-bee pollen from Türkiye and Slovenia showed almost identical patterns (Figure 5A), and, additionally, they had similar profiles as their botanical source *C. sativa* (Figure 5D), especially at R_F_ values between ≈ 0.55 and 0.85. S-bee pollen from Türkiye and Slovenia showed almost identical HPTLC patterns (Figure 5B). The pattern of the three characteristic zones at R_F_ ≈ 0.3 to 0.5 was only observed in S-bee pollen (Figure 5B) but not in C-bee pollen (Figure 5A) and Q-bee pollen (Figure 5C). HPTLC fingerprints of *S. alba* androecia and S-bee pollen (Figure 5E) were nearly identical. Q-bee pollen (Figure 5C) and *Q. pubescens* androecia (Figure 5F) had very similar HPTLC profiles.

Among the botanical sources studied (*C. sativa*, *S. alba*, and *Q. pubescens*), *C. sativa* is the only species in the *Castanea* genus found in both countries (Türkiye and Slovenia). In addition to *S. alba* and *Q. pubescens*, other species belonging to *Salix* spp. and *Quercus* spp. should be studied to obtain more information about the botanical sources of bee pollen.

**Figure 6:**
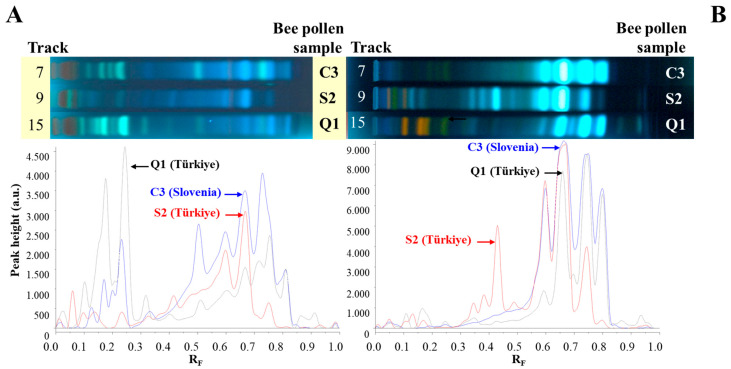
HPTLC fingerprints of C-bee pollen (C3), S-bee pollen (S2), and Q-bee pollen (Q1) based on chromatograms at 366 nm after using anisaldehyde (**A**) and NP/PEG (**B**) reagents and videodensitogram profiles obtained from chromatograms (Figure 4A,C) by image analysis in fluorescence mode. Application amounts of all samples per band: 0.1 mg.

HPTLC fingerprints based on chromatograms at 366 nm after using anisaldehyde (Figure 6A) and NP/PEG (Figure 6B) reagents and videodensitogram profiles obtained from them by image analysis in fluorescence mode enabled the distinction between bee pollen samples from different botanical sources. Figure 6A,B show a comparison of the profiles of C3 and S2 bee pollen samples having different botanical sources with the highest dominant pollen grains (Table 1) and Q1 bee pollen after using anisaldehyde and NP/PEG at 366 nm, respectively. It is evident that different derivatization reagents provide a wide range of information about zones for compounds which can be used as identification markers.

### 3.3. UPLC Analyses

A newly developed UPLC-PDA method was first validated using the parameters as follows: specificity, linearity, recovery, intraday and interday precision, limit of detection (LOD), and limit of quantification (LOQ). This method was used for the quantification of the compounds *N*^1^,*N*^5^,*N*^10^-tricaffeoylspermidine, rutin, myricitrin, hyperoside, isoquercitrin, quercitrin, and quercetin in bee pollen and androecia samples.

#### 3.3.1. UPLC Method Validation

##### Specificity

**Figure 7:**
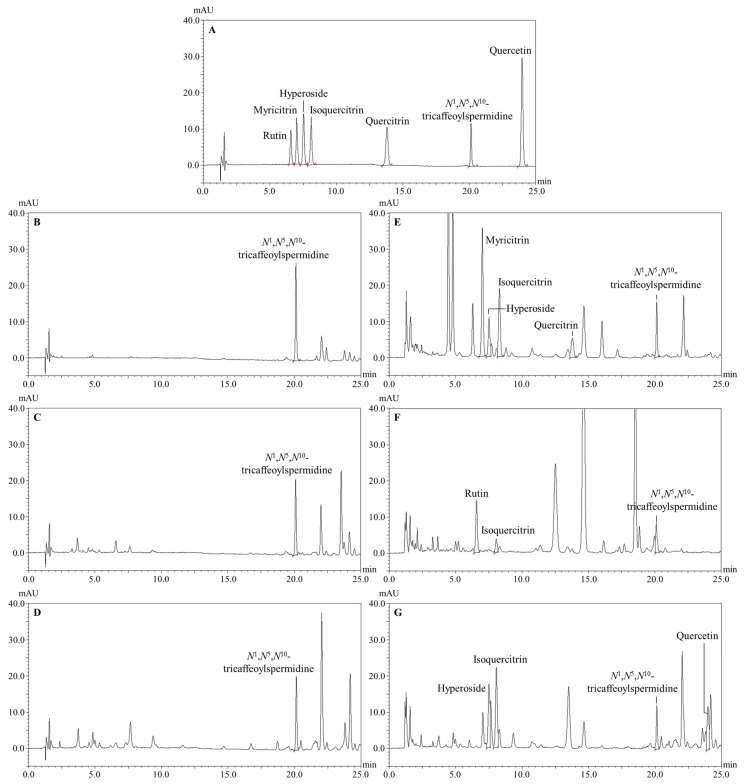
UPLC chromatograms of compounds investigated (**A**), C-bee pollen—C1 (**B**), S-bee pollen—S1 (**C**), Q-bee pollen—Q1 (**D**), *C. sativa* androecia (**E**), *S. alba* androecia (**F**), and *Q. pubescens* androecia (**G**) at 256 nm (**A**,**E**–**G**) and 320 nm (**B**–**D**).

The compounds investigated were identified by comparing the retention times (t_R_) and UV spectra of the compounds evaluated in bee pollen and androecia samples with the t_R_ and UV spectra of compounds in the standard mixture (Figure 7). Retention times of the compounds investigated were found as follows: rutin (t_R_ ≈ 6.60); myricitrin (t_R_ ≈ 7.05); hyperoside (t_R_ ≈ 7.56); isoquercitrin (t_R_ ≈ 8.13); quercitrin (t_R_ ≈ 13.85); *N*^1^,*N*^5^,*N*^10^-tricaffeoylspermidine (t_R_ ≈ 20.15); and quercetin (t_R_ ≈ 24.01) (Figure 7). Since specificity is the ability of the method to measure only the compound of interest without being influenced by other components that may be present in the sample, the evaluation of this parameter was based on whether or not peaks of the respective standards are detected in the blank chromatogram. As a result of this evaluation, chromatographic peaks belonging to *N*^1^,*N*^5^,*N*^10^-tricaffeoylspermidine, rutin, myricitrin, hyperoside, isoquercitrin, quercitrin, and quercetin were not detected in the blank chromatogram, which confirmed the specificity of the method.

##### Linearity of the Calibration Curve, Limit of Detection (LOD), and Limit of Quantification (LOQ)

Calibration curves of the investigated standards were obtained by plotting the average peak areas obtained after analyzing standard solutions with seven different concentrations ranging from 0.5 to 50 µg/mL in triplicate. The coefficient of determination values (r^2^) for calibration curves of each of the standards were over 0.99, showing a good linear correlation (Table 4). LOD and LOQ values were determined using the equations 3 × (SD/S) and 10 × (SD/S), respectively (Table 4).

##### Precision

Intraday and interday precision is the degree of agreement between repeated measurements of the same sample with the developed method and were determined by analyzing 5 µg/mL of all standards at different times of the same day and on two different days, respectively. The results were expressed with relative standard deviation (RSD) values (Table 5). The RSD values were found in the range of 0.069–0.891 for intraday precision and 0.015–0.678 for interday precision. As recommended in [39], precision results should be ≤5%. The values obtained (Table 5) meet these criteria, indicating the sensitivity of the method.

##### Accuracy

Accuracy of the developed method was evaluated by the percentage recovery and RSD values of the found amounts of standards at three concentrations as 3, 6, and 12 μg/mL using the calibration curves. The results obtained were evaluated as recovery.

The results were found to be 93.9–98.4% for *N*^1^,*N*^5^,*N*^10^-tricaffeoylspermidine, 98.4–103.4% for rutin, 95.5–99.0% for myricitrin, 96.8–102.9% for hyperoside, 98.2–101.2% for isoquercitrin, 102.0–105.4% for quercitrin, and 94.2–98.7% for quercetin (Table 6). As a consequence, the accuracy of the developed method was determined based on the recovery percentages and was found to be within a recommended acceptable range of 80–120% [40].

#### 3.3.2. Quantitative Analyses

The UPLC method developed and validated in this study was used for the quantitation of the main compounds in both bee pollen and androecia samples. The results are hereby presented in Table 7 as mg of the compound investigated per hydroalcoholic bee pollen and androecia extracts (mg/g). 

*N*^1^,*N*^5^,*N*^10^-tricaffeoylspermidine was the main compound in bee pollen samples (from Türkiye and Slovenia; ranging between ≈3 mg/g and 41 mg/g) (Table 7). Among the bee pollen samples, the highest content of *N*^1^,*N*^5^,*N*^10^-tricaffeoylspermidine was found in C-bee pollen (ranging between ≈26 mg/g and 41 mg/g) (Table 7). In addition to *N*^1^,*N*^5^,*N*^10^-tricaffeoylspermidine, other spermidine derivatives such as *N*^1^,*N*^5^,*N*^10^-tricoumaroylspermidine, *N*^1^,*N*^10^-dicoumaroyl-*N*^5^-caffeoylspermidine, *N*^1^-coumaroyl-*N*^5^,*N*^10^-dicaffeoylspermidine, and *N*^1^-feruloyl-*N*^5^,*N*^10^-dicaffeoylspermidine were found in bee pollen samples originating from *C. sativa* [33].

As in this study, *N*^1^,*N*^5^,*N*^10^-tricaffeoylspermidine was reported as the dominant compound in ethanolic extracts of *C. sativa*-originated bee pollen samples from Italy [41] and the northwest Iberian Peninsula (Galicia and North of Portugal) [42]. Previous studies [41,42], as well as this study, indicate that the main compound(s) found in bee pollen are independent of the collection sites and periods (years) but dependent on the botanical origin of the bee pollen samples. Based on the results obtained in these studies, food supplements containing bee pollen originating from *C. sativa* could be standardized over *N*^1^,*N*^5^,*N*^10^-tricaffeoylspermidine.

*N*^1^,*N*^5^,*N*^10^-tricaffeoylspermidine was quantified in S-bee pollen (ranging from ≈5.5 to 21 mg/g) and Q-bee pollen (≈3 mg/g) (Table 7). Quantitative data obtained for *N*^1^,*N*^5^,*N*^10^-tricaffeoylspermidine in S-bee pollen (Table 7) and the highest intensity of the zones (R_F_ ≈ 0.67) in HPTLC chromatograms (Figure 2D, tracks 5–11) confirmed that *N*^1^,*N*^5^,*N*^10^-tricaffeoylspermidine was the major compound, suggesting that S-bee pollen-containing dietary supplements could also be standardized via *N*^1^,*N*^5^,*N*^10^-tricaffeoylspermidine. In the case of Q-bee pollen, more samples should be investigated, and other main compounds at R_F_ 0.74 and 0.81 should be identified (Figure 2D, track 11). Parallel to this study, spermidine derivatives were identified in bee pollen originating from *Q. mongolica* [34]. *N*^1^,*N*^5^,*N*^10^-tricaffeoylspermidine was also found to be a common compound in the androecia of *C. sativa* (≈6 mg/g), *S. alba* (≈3.5 mg/g), and *Q. pubescens* (≈5 mg/g) (Table 7). To the best of our knowledge, this is the first study identifying and quantifying *N*^1^,*N*^5^,*N*^10^-tricaffeoylspermidine in the androecia of *C. sativa*, *S. alba*, and *Q. pubescens*. *N*^1^,*N*^5^,*N*^10^-tricaffeoylspermidine and other spermidine derivatives such as *N*^1^-*p*-coumaroyl-*N*^5^,*N*^10^-dicaffeoylspermidine, *N*^1^,*N*^10^-di-*p*-coumaroyl-*N*^5^-caffeoylspermidine, and *N*^1^,*N*^5^,*N*^10^-tri-*p*-coumaroylspermidine were identified in the flower pollen of *Q. dentata,* but their quantity was not reported [19]. Rutin (quercetin-3-*O*-rutinoside) was found in the androecia of *S. alba* (0.6 mg/g) and *Q. pubescens* (0.5 mg/g) but not in *C. sativa* (Table 7). To the best of our knowledge, this is the first study identifying rutin in the androecia of *S. alba* and *Q. pubescens*. Rutin was also identified in *S. aegyptiaca* catkins [15]. In this study, rutin was found in C-bee pollen and S-bee pollen by HPTLC (Figure 2) but was quantified by UPLC only in Q-bee pollen (≈1 mg/g) (Table 7), possibly because different concentrations were used for HPTLC and UPLC analyses. In another study, rutin (≈0.18 mg/g) was detected in a methanolic extract of bee pollen originating from *C. sativa* [43]. Myricitrin (myricetin-3-*O*-rhamnoside) was quantified in the androecia of *C. sativa* (≈16 mg/g), but it was not detected in other androecia samples (Table 7). To the best of our knowledge, this is the first study identifying myricitrin in the androecia of *C. sativa*. Myricetin derivatives such as myricetin-3-*O*-glucuronide (≈0.36 mg/g) and myricetin-3-*O*-glucoside (≈0.38 mg/g) were determined in the hydroalcoholic extract of *C. sativa* catkins collected in Portugal, but myricitrin was not detected [44]. Myricetin-3-*O*-glucuronide (*judia* cultivar ≈ 1 mg/g, *longal* cultivar ≈ 0.1 mg/g) and myricetin-3-*O*-glucoside (*judia* cultivar ≈ 0.6 mg/g, *longal* cultivar ≈ 0.1 mg/g) were found in extracts of lyophilized *C. sativa* flowers (both cultivars) prepared with infusion and decoction techniques [45]. Hyperoside (quercetin-3-*O*-galactoside) was found in the androecia of *C. sativa* (≈4 mg/g) and *Q. pubescens* (≈7 mg/g) but was not detected in the androecia of *S. alba* (Table 7). To the best of our knowledge, this is the first study identifying hyperoside in the androecia of *C. sativa* and *Q. pubescens*. Isoquercitrin was found to be a common component in the analyzed androecia samples, with the highest content in *Q. pubescens* (≈11 mg/g) and the lowest content in *S. alba* (≈2 mg/g) (Table 7). To the best of our knowledge, this is the first study identifying isoquercitrin in the androecia of *Q. pubescens* and *S. alba*. Isoquercitrin content (≈9 mg/g) in the androecia of *C. sativa* (Table 7) was much higher than the contents reported for the samples collected in Portugal: ≈0.9 mg/g in the hydroalcoholic extract of *C. sativa* catkins [44]; ≈2 mg/g in extracts prepared with a decoction technique from lyophilized *C. sativa* flowers (*judia* cultivar) [45]. Isoquercitrin was not detected in bee pollen samples (Table 7). However, it was found in the ethyl acetate sub-extract of bee pollen originating from *S. alba* (≈0.7 mg/g) [46]. Quercitrin (quercetin-3-*O*-rhamnoside) was only found in *C. sativa* androecia (≈3 mg/g) (Table 7), and it was reported also in a hydroalcoholic extract of *C. sativa* catkins from Portugal but in a much lower amount (0.1 mg/g) [44]. Quercetin was only quantified in the androecia of *Q. pubescens* (≈2.5 mg/g) but was not detected in other samples (Table 7). To the best of our knowledge, this is the first study identifying and quantifying quercetin in *Q. pubescens* androecia. Although quercetin was detected in *Quercus* spp. bee pollen by HPTLC (Figure 2), it was not quantified by UPLC (Table 7), which could be a consequence of different concentrations used for HPTLC and UPLC analyses.

The variations in chemical composition could be because of growth conditions such as frequency of the rain fall, soil pH, and naturally grown and cultivar trees. In addition, the plant parts used in all these studies such as the androecia and flowers (male or female) may differ in chemical composition. Some studies [44,45] did not specify the type of flowers (male or female), therefore, the terminology from these studies (catkins [15,45], flowers [44]) was used throughout this discussion.

### 3.4. In Vitro Bioactivity Analyses

#### 3.4.1. Antioxidant Activity Determined by DPPH, FRAP, ABTS, and CUPRAC Assays

The antioxidant activities of bee pollen, androecia samples, and *N*^1^,*N*^5^,*N*^10^-tricaffeoylspermidine were evaluated using DPPH, FRAP, ABTS, and CUPRAC assays. *N*^1^,*N*^5^,*N*^10^-tricaffeoylspermidine showed the highest antioxidant activity. This is the first report to evaluate the antioxidant activity of *N*^1^,*N*^5^,*N*^10^-tricaffeoylspermidine. Among all the bee pollen samples, the samples with high content of *N*^1^,*N*^5^,*N*^10^-tricaffeoylspermidine (Table 7) as C-bee pollen containing 85–98% of *C. sativa* flower pollen (Table 1) showed higher antioxidant activity than the S-bee pollen and Q-bee pollen (Table 8). When the bee pollen samples from *Salix* spp. were compared to each other, S2 and S7 (containing 89.8% and 87.8% *Salix* spp. flower pollen, respectively (Table 1)), having higher *N*^1^,*N*^5^,*N*^10^-tricaffeoylspermidine content (Table 7), exerted higher antioxidant activity than the other samples (Table 8). Q-bee pollen with 53.8% of *Quercus* spp. flower pollen (Table 1) and the lowest *N*^1^,*N*^5^,*N*^10^-tricaffeoylspermidine content (Table 7) showed the lowest antioxidant activity (Table 8).

All antioxidant assays (DPPH, FRAP, CUPRAC, and ABTS) proved *N*^1^,*N*^5^,*N*^10^-tricaffeoylspermidine (Table 8) to be a more potent antioxidant than phenolic acids (chlorogenic acid and 3,5-dicaffeoylquinic acid) and flavonoids (afzelin, platanoside, and quercetin-3-*O*-β-glucopyranosyl-(1→2)-β-galactopyranoside) [10]. Similar findings about phenolamines (di-*p*-coumaroyl spermidine, di-*p*-coumaroyl hydroxyferuloyl spermine, tri-*p*-coumaroyl spermidine, etc.) having higher antioxidant activity than flavonoids (quercetin and kaempferol derivatives) were also reported in another study [4]. In addition, it was reported that phenolamides (di-*p*-coumaroylspermidine, tri-*p*-coumaroylspermidine, and tetra-*p*-coumaroylspermine) had a positive correlation with the antioxidant activity of rape bee pollen [32].

As reported in the literature, bee pollen originating from *C. sativa*, *Salix* spp., and *Quercus* spp. were extracted using different extraction procedures, and the antioxidant activity of the extracts was evaluated using different methods. Other studies of bee pollen samples originating from *C. sativa* reported values of 17.08–38.56 mg TE/g by ABTS assay [47] and 20.24 mg TE/g by FRAP assay [48]. For bee pollen originating from *Salix* spp., values of 18.66 mg TE/g, 75.64 mg TE/g, and 9.92 TE/g were reported, respectively, by DPPH, CUPRAC, and FRAP assays [46].

In *C. sativa*, *S. alba*, and *Q. pubescens* androecia samples, not only *N*^1^,*N*^5^,*N*^10^-tricaffeoylspermidine but also other compounds may contribute to the antioxidant activity. Among the androecia samples, *C. sativa* had the highest antioxidant activity (Table 8).

#### 3.4.2. Xanthine Oxidase Inhibitory Activity

The XO inhibitory activity was evaluated in *N*^1^,*N*^5^,*N*^10^-tricaffeoylspermidine, bee pollen, and androecia samples using allopurinol as a positive control (IC_50_ = 3.07 µg/mL). A significant XO inhibitory activity was obtained for *N*^1^,*N*^5^,*N*^10^-tricaffeoylspermidine (IC_50_ = 2.98 µg/mL) (Table 9). This is the first study evaluating the XO inhibitory activity of *N*^1^,*N*^5^,*N*^10^-tricaffeoylspermidine. Among bee pollen samples, C-bee pollen had the highest XO inhibitory activity (with IC_50_ values ranging from 26.18 to 31.52 µg/mL (Table 9)) that could be related to *N*^1^,*N*^5^,*N*^10^-tricaffeoylspermidine contents, which were higher than in other bee pollen samples (Table 7). Similarly, among five bee pollen fractions, the fraction with coumaroylspermidines showed higher XO inhibitory activity, suggesting that spermidines can be considered as the components responsible for this activity [49]. Among androecia samples, the highest XO inhibitory activity was observed for *C. sativa* (IC_50_ = 15.97 µg/mL), the next highest was for *Q. pubescens* (IC_50_ values 20.83 µg/mL), and the lowest was for *S. alba* (IC_50_ = 23.74 µg/mL) (Table 9). In addition to *N*^1^,*N*^5^,*N*^10^-tricaffeoylspermidine, other phenolic compounds may contribute to the XO inhibitory activity.

### 3.5. HPTLC-Effect-Directed Analyses

#### 3.5.1. HPTLC-DPPH Analyses of Antioxidant Activity

**Figure 8:**
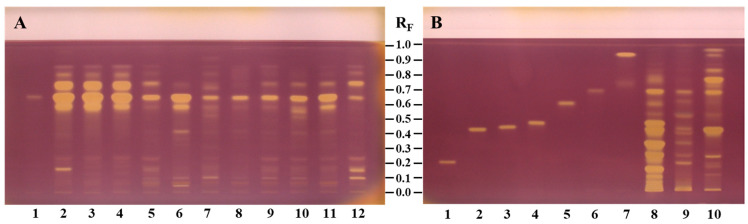
HPTLC silica gel NH_2_ F_254S_ plate developed with ethyl acetate–formic acid–water (35:4:4, *v*/*v*/*v*), documented at white light after detection with DPPH reagent. Applications per band: (**A**) track 1: *N*^1^,*N*^5^,*N*^10^-tricaffeoylspermidine (0.4 μg); track 2–4: C-bee pollen (C1–C3, 0.1 mg); track 5–11: S-bee pollen (S1–S7, 0.1 mg); track 12: Q-bee pollen (Q1, 0.1 mg). (**B**) track 1: rutin (0.4 μg); track 2: hyperoside (0.4 μg); track 3: isoquercitrin (0.4 μg); track 4: myricitrin (0.4 μg); track 5: quercitrin (0.4 μg); track 6: *N*^1^,*N*^5^,*N*^10^-tricaffeoylspermidine (0.4 μg); track 7: quercetin (0.4 μg); track 8: *C. sativa* androecia (0.1 mg); track 9: *S. alba* androecia (0.1 mg); track 10: *Q. pubescens* androecia (0.1 mg).

**Figure 9:**
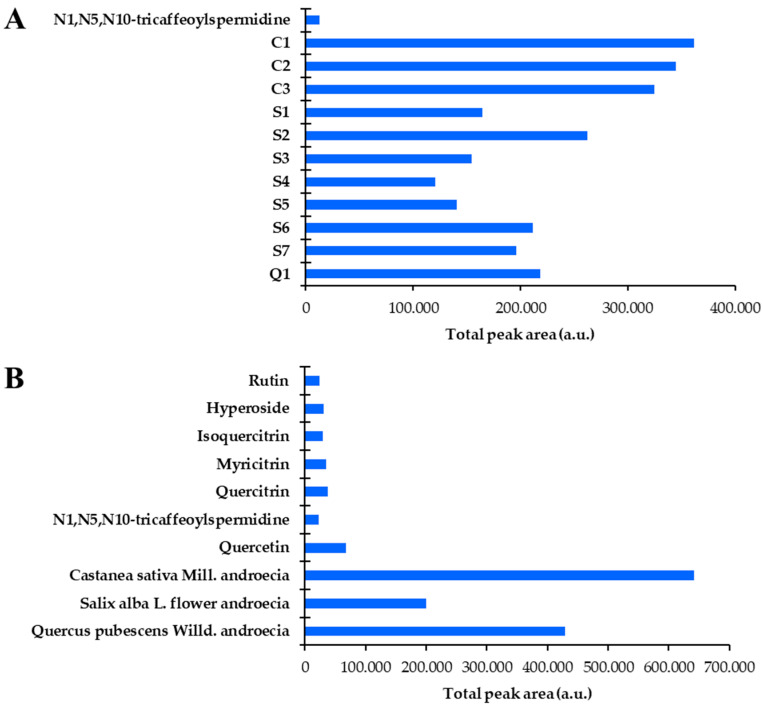
Comparison of total peak areas of all yellow bands detected in the videodensitograms obtained by image analysis of HPTLC-DPPH chromatograms (Figure 8A,B) in fluorescent mode. (**A**) Total peak areas of *N*^1^,*N*^5^,*N*^10^-tricaffeoylspermidine and bee pollen samples. (**B**) Total peak areas of standards investigated and androecia samples.

In addition to the *in vitro* antioxidant analyses using spectrophotometry, HPTLC-DPPH analyses of the investigated compounds, bee pollen, and androecia samples were performed to obtain additional information on the antioxidant activity of compounds separated on the HPTLC plate. Figure 8 shows the HPTLC plates with yellow-colored chromatographic zones which indicate compounds that have antioxidant activity; the images of the plates were captured under white light after 30 min. Zones at the R_F_ of *N*^1^,*N*^5^,*N*^10^-tricaffeoylspermidine were more intense in C-bee pollen than in other bee pollen samples (Figure 8A). C-bee pollen had higher contents of *N*^1^,*N*^5^,*N*^10^-tricaffeoylspermidine than S-bee pollen and Q-bee pollen (Table 6). C-bee pollen had higher antioxidant potential than S-bee pollen and Q-bee pollen (Figure 9A). These results were found to be in parallel with *in vitro* assays (Table 7). As illustrated in Figure 9A, not only *N*^1^,*N*^5^,*N*^10^-tricaffeoylspermidine but apparently also other compounds separated on the chromatogram contribute to the antioxidant effect. The total peak areas of the yellow zones showed that C-bee pollen had the highest antioxidant activity (Figure 9A).

Evaluation of *N*^1^,*N*^5^,*N*^10^-tricaffeoylspermidine and six other compounds (rutin, hyperoside, isoquercitrin, myricitrin, quercitrin, and quercetin, all having the same 0.4 μg application amounts) demonstrated that the intensity of the yellow zones belonging to flavonoids was higher than *N*^1^,*N*^5^,*N*^10^-tricaffeoylspermidine (Figure 8B).

In androecia samples (Figure 8B, tracks 8–10), several yellow zones were observed showing the antioxidant activity contributions of the compounds investigated as well as several other yet unidentified compounds. In particular, these intense yellow zones were observed from the application position at R_F_ ≈ 0.5 in *C. sativa* androecia, showing a high contribution to the antioxidant activity (Figure 8B, track 8). Based on the total peak areas of the yellow zones, *C. sativa* androecia had the highest antioxidant activity (Figure 9B).

#### 3.5.2. HPTLC-XO Inhibitory Activity

**Figure 10:**
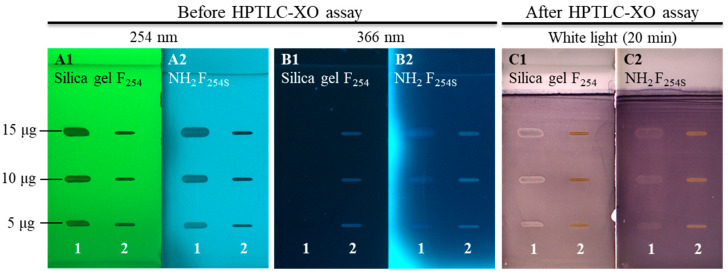
Comparison of HPTLC-XO assay on HPTLC silica gel F_254_ and HPTLC NH_2_ F_254S_ plates using allopurinol (positive control, track 1) and *N*^1^,*N*^5^,*N*^10^-tricaffeoylspermidine (track 2) applied on the plates after development with ethyl acetate–formic acid–water (35:4:4, *v*/*v*/*v*). Plates were documented before HPTLC-XO assay at 254 nm (**A1**,**A2**) and 366 nm (**B1**,**B2**) and under white light (20 min) after the HPTLC-XO assay (**C1**,**C2**).

**Figure 11:**
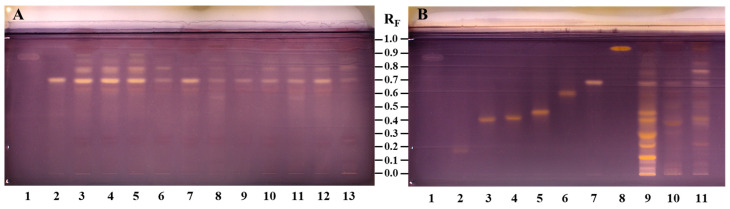
HPTLC NH_2_ F_254S_ plate developed with ethyl acetate–formic acid–water (35:4:4, *v*/*v*/*v*), documented under white light after HPTLC-XO assay. Applications per band: (**A**) track 1: allopurinol (15 µg); track 2: *N*^1^,*N*^5^,*N*^10^-tricaffeoylspermidine (5 μg); tracks 3–5: C-bee pollen (C1–C3, 0.25 mg); tracks 6–12: S-bee pollen (S1–S7, 0.25 mg); track 13: Q-bee pollen (Q1, 0.25 mg). (**B**) track 1: allopurinol (15 µg); track 2: rutin (5 μg); track 3: hyperoside (5 μg); track 4: isoquercitrin (5 μg); track 5: myricitrin (5 μg); track 6: quercitrin (5 μg); track 7: *N*^1^,*N*^5^,*N*^10^-tricaffeoylspermidine (5 μg); track 8: quercetin (5 μg); track 9: *C. sativa* androecia (0.25 mg); track 10: *S. alba* androecia (0.25 mg); track 11: *Q. pubescens* androecia (0.25 mg).

To supplement the results of the spectrophotometric XO inhibitory activity assay of the whole hydroalcoholic extract of each of the bee pollen and androecia samples and *N*^1^,*N*^5^,*N*^10^-tricaffeoylspermidine (Table 9), the XO inhibitory activity of the samples and standards was analyzed by an HPTLC-XO assay providing more information about the XO inhibitors separated on the HPTLC plate. The XO inhibitors were detected as white/yellow zones on a purple background (Figure 10 and Figure 11). This is the first study performing HPTLC-XO inhibitory activity analysis on the HPTLC NH_2_ F_254S_ plates (Figure 10 and Figure 11).

To understand the applicability of the HPTLC-XO inhibitory activity on HPTLC NH_2_ F_254S_ plates, a comparative analysis was performed with HPTLC silica gel F_254_ plates. Both types of plates were first developed with ethyl acetate–formic acid–water (35:4:4, *v*/*v*/*v*). After development, the plates were dried in a stream of cold air, followed by separate applications of 5 µg, 10 µg, and 15 µg of allopurinol (positive control) (Figure 10, track 1) and *N*^1^,*N*^5^,*N*^10^-tricaffeoylspermidine (Figure 10, track 2), followed by documentation of the images of the plates at 254 nm (Figure 10A1,A2) and 366 nm (Figure 10B1,B2). The HPTLC-XO inhibitory activity assay was then performed as described in the Section 2.9.2. HPTLC-xanthine oxidase inhibitory activity and plates were documented immediately (t = 0 min) and after 5, 10, 15, 20, 30, 45, 60, 90, and 120 min under white light (Figure S4). Between intervals, the plates were stored in the dark. Figure 10C1,C2 show the plates under white light (20 min) after HPTLC-XO assay.

Several differences were observed between HPTLC-XO inhibitory activity assays on silica and amino plates during the 120 min time interval. It is evident that the stationary phase influences the detectability of both compounds on the background which becomes darker in the time interval used (0 to 120 min) (Figure S4). The background was pale purple for the silica gel plate and dark purple for the amino plate at the beginning of the documentation (t = 0 min) (Figure S4). At the beginning (t = 0 min), it was easier to detect the white zone with the lowest amount of allopurinol (5 µg) on the amino plate than on the silica gel plate. However, after 15 min, the same amount of allopurinol (5 µg) was easier to detect on the silica gel plate than on the amino plate because the background became darker (Figure S4, track 1). *N*^1^,*N*^5^,*N*^10^-tricaffeoylspermidine was observed as a yellow zone on silica gel and amino plates (Figure S4, track 2). Intensive yellow bands for *N*^1^,*N*^5^,*N*^10^-tricaffeoylspermidine and additional light frames around the *N*^1^,*N*^5^,*N*^10^-tricaffeoylspermidine zone were observed from 15 to 120 min on the silica gel plate. On the amino plate, a yellow *N*^1^,*N*^5^,*N*^10^-tricaffeoylspermidine zone was clearly observed at the beginning, and its intensity was increasing during the time intervals studied (Figure S4, track 2).

Based on these observations, HPTLC-XO inhibitory activity results for allopurinol (positive control), bee pollen, androecia samples, *N*^1^,*N*^5^,*N*^10^-tricaffeoylspermidine, rutin, hyperoside, isoquercitrin, myricitrin, quercitrin, and quercetin were given after 20 min (Figure 11). The yellow-white zone of *N*^1^,*N*^5^,*N*^10^-tricaffeoylspermidine (at R_F_ ≈ 0.67, Figure 11A, track 2 and Figure 11B, track 7) was detected in all bee pollen (Figure 11A, tracks 3–13) and androecia samples (Figure 11B, tracks 9–11). Among bee pollen samples, C-bee pollen (Figure 11A, tracks 3–5) showed the highest number of yellow-white zones. Additionally, the C-bee pollen (Figure 11A, tracks 3–5) gave more intense yellow-white zones at R_F_ ≈ 0.67 (*N*^1^,*N*^5^,*N*^10^-tricaffeoylspermidine) and between R_F_ ≈ 0.74 and ≈ 0.85 (Figure 11A, tracks 3–5) than S-bee pollen (Figure 11A, tracks 6–12) and Q-bee pollen (Figure 11A, tracks 13). Among the S-bee pollen, S2 and S7, with higher of *N*^1^,*N*^5^,*N*^10^-tricaffeoylspermidine content (Table 7), showed more intense yellow-white zones (Figure 11A, tracks 7 and 12) than other samples. This is in agreement with the results of the spectrophotometric assay (Table 9). Among the standards investigated, flavonoids were observed as yellow zones (Figure 11B, tracks 2–6 and 8). Among the androecia samples, *C. sativa* androecia (Figure 11B, track 9) showed the highest number of yellow zones compared to *S. alba* (Figure 11B, track 10) and *Q. pubescens* androecia (Figure 11B, track 11), which is in agreement with the results of the spectrophotometric assay (Table 9).

## 4. Conclusions

This is the first study comparing the phytochemical composition and bioactivity profiles of bee pollen originating from *C. sativa*, *Salix* spp., and *Quercus* spp. from Türkiye and Slovenia and the androecia of *C. sativa*, *S. alba*, and *Q. pubescens.* Comparisons were based on newly developed qualitative HPTLC and quantitative UPLC methods. *N*^1^,*N*^5^,*N*^10^-tricaffeoylspermidine, the compound common to bee pollen in this study, was found to have the highest content in C-bee pollen. In addition, *N*^1^,*N*^5^,*N*^10^-tricaffeoylspermidine was for the first time identified and quantified in S-bee pollen, where it was found in relatively high content levels. *N*^1^,*N*^5^,*N*^10^-tricaffeoylspermidine was for the first time identified and quantified in *C. sativa*, *S. alba*, and *Q. pubescens* androecia, where it was also found as a common compound. Among the C-, S-, and Q-bee pollen, bee pollen having the same botanical origin showed identical chemical fingerprint patterns by HPTLC image analysis showing that bee pollen composition is not related to the collection location, geographical differences, and the bee race. HPTLC profiles of bee pollen samples showed similarities and differences at specified R_F_ ranges when compared with profiles of the relevant botanical sources. The similarities helped with the identification of the botanical sources, but attention should be drawn to the reasons for the differences. The reason for these differences may be the morphological and chemical changes that flower pollen undergoes during pellet formation. Furthermore, since *C. sativa* is the only species in the genus *Castanea* found in both countries (Türkiye and Slovenia), other *Salix* and *Quercus* species should also be investigated in terms of chemical composition to find out if they could be a potential pollen grain source.

The antioxidant activity and XO inhibition of bee pollen, androecia, and *N*^1^,*N*^5^,*N*^10^-tricaffeoylspermidine were evaluated by *in vitro* tests and HPTLC-EDA. HPTLC-EDA, which provided the detection of bioactive zones on the plate, was combined with image analysis that enabled the evaluation of bee pollen and androecia together with *N*^1^,*N*^5^,*N*^10^-tricaffeoylspermidine, quercetin, myricitrin, hyperoside, quercitrin, isoquercitrin, and rutin. *N*^1^,*N*^5^,*N*^10^-tricaffeoylspermidine had remarkable antioxidant and XO inhibitory activities, which were evaluated for the first time by HPTLC-EDA and *in vitro* tests. This is the first report on performing an HPTLC-XO inhibitory activity assay on the HPTLC NH_2_ F_254S_ plates and evaluating their suitability related to commonly used HPTLC silica gel F_254_ plates.

The high content and bioactivity of *N*^1^,*N*^5^,*N*^10^-tricaffeoylspermidine demonstrate its potential to be used as a marker compound for the standardization of bee pollen extracts. Overall, botanically identified and standardized bee pollen extracts should be used in food supplements to ensure repeatable pharmacological activity.

## Figures and Tables

**Table 1:** Botanical origins and locations of collected bee pollen samples.

Bee Pollen Samples	Sample Codes	Botanical Sources (Dominant Pollen (%))	Locations
C-bee pollen	C1	*Castanea sativa* (85.8%)	İzmit (Türkiye)
	C2	*Castanea sativa* (97.0%)	Artvin (Türkiye)
	C3	*Castanea sativa* (98.0%)	Ptuj (Slovenia)
S-bee pollen	S1	*Salix* spp. (52.8%)	Ordu (Türkiye)
	S2	*Salix* spp. (89.8%)	Giresun (Türkiye)
	S3	*Salix* spp. (45.1%)	Çanakkale (Türkiye)
	S4	*Salix* spp. (56.7%)	Begunje na Gorenjskem (Slovenia)
	S5	*Salix* spp. (62.1%)	Žirovnica (Slovenia)
	S6	*Salix* spp. (63.5%)	Kranj (Slovenia)
	S7	*Salix* spp. (87.8%)	Ljubljana (Slovenia)
Q-bee pollen	Q1	*Quercus* spp. (53.8%)	Ankara (Türkiye)

**Table 2:** The structure and NMR data (^13^C (100 MHz) and ^1^H (400 MHz)) of *N*^1^,*N*^5^,*N*^10^-tricaffeoylspermidine (DMSO).

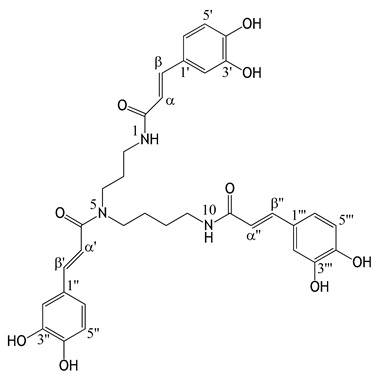		
	C_34_H_37_N_3_O_9_	
	Mol. Wt.: 631.7	
**Position**	***δ*_C_** **(ppm)**	***δ*_H_** **(ppm), *J* (Hz)**
2	36.7	3.47 m
3	27.3	1.42–1.72 m
4	45.9	3.19 ^ϯ^
6	47.5	3.19 ^ϯ^
7	27.0 ^ϯ^	1.42–1.72 m
8	27.0 ^ϯ^	1.42–1.72 m
9	37.0	3.36 m
**Tricaffeoyl (*N*^1^,*N*^5^,*N*^10^)**		
α/α′/α″	116.1	6.33 d, (15.9)
β/β′/β″	146.5	7.24 d, (15.9)
1′/1″/1‴	126.3	-
2′/2″/2‴	113.9	6.94 ^ϯ^
3′/3″/3‴	139.7	-
4′/4″/4‴	142.7	-
5′/5″/5‴	116.1	6.70–6.79 ^ϯ^
6′/6″/6‴	120.8	6.70–6.79 ^ϯ^
3-CO	165.9	-

^ϯ^ Overlapped signals.

**Table 3:** Common chromatographic zones in C-bee pollen (C1–C3), S-bee pollen (S1–S7), Q-bee pollen (Q1), and androecia of *C. sativa* (C-a), *S. alba* (S-a), and *Q. pubescens* (Q-a).

		Samples													
Reagent	R_F_ values/Color	C-a	C1	C2	C3	S-a	S1	S2	S3	S4	S5	S6	S7	Q-a	Q1
NP/PEG (366 nm)	0.07/Green	-	-	-	-	+	+	+	+	+	+	+	+	-	-
	0.11/Orange	-	+	-	-	-	+	+	+	+	+	+	+	+	+
	0.16/Orange	-	+	-	-	-	+	-	+	-	+	+	+	+	+
	0.19/Orange	-	-	-	-	+	+	-	+	-	+	+	+	+	+
	0.22/Green	-	+	+	+	-	-	-	-	-	-	-	-	-	-
	0.25/Green	-	+	+	+	+	+	-	+	-	+	+	+	+	+
	0.35 /Light blue	-	-	-	-	+	+	+	+	+	+	+	+	-	-
	0.38/Light blue	-	-	-	-	-	+	+	+	+	+	+	+	-	-
	0.43/Light blue	-	-	-	-	+	+	+	+	+	+	+	+	-	-
	0.6/Light blue	+	+	+	+	+	+	+	+	+	+	+	+	+	+
	0.67/Light blue (*N*^1^,*N*^5^,*N*^10^-tricaffeoylspermidine)	+	+	+	+	+	+	+	+	+	+	+	+	+	+
	0.70/Light blue	-	-	-	-	-	+	-	-	-	+	+	+	+	+
	0.74/Light blue	+	+	+	+	+	+	+	+	+	+	+	+	+	+
	0.81/Light blue	+	+	+	+	+	+	-	+	-	+	+	+	+	+
ANIS (white light)	0.07/Brown	+	+	+	+	+	+	+	+	+	+	+	+	+	+
	0.61/Violet	+	+	+	+	+	+	+	+	+	+	+	+	+	+
	0.72/Yellow	+	+	+	+	-	-	-	-	-	-	-	-	+	+
	0.77/Blue	+	+	+	+	-	-	-	-	-	-	-	-	+	+
	0.84/Blue	-	-	-	-	-	+	-	-	-	+	+	-	+	+
	0.89/Red-Brown	-	-	-	-	+	-	-	+	-	-	-	-	-	-
	0.91/Violet	-	-	-	-	-	-	-	-	-	-	-	-	+	+
ANIS (366 nm)	0.07/Black-Brown	+	+	+	+	+	+	+	+	+	+	+	+	+	+
	0.11/Green	-	-	-	-	+	+	+	+	+	+	+	+	+	+
	0.19/Green	-	+	+	+	+	+	-	+	-	+	+	+	+	+
	0.22/Green	+	+	+	+	-	-	-	-	-	-	-	-	+	+
	0.25/Green	-	+	+	+	+	+	-	+	-	+	+	+	+	+
	0.52/Green-Blue	+	+	+	+	+	+	-	+	-	+	+	-	+	+
	0.61/Blue	-	+	+	+	+	-	+	-	-	-	-	+	-	-
	0.63/Red	-	+	+	+	+	+	+	+	+	+	+	+	-	+
	0.67/Blue (*N*^1^,*N*^5^,*N*^10^-tricaffeoylspermidine)	+	+	+	+	+	+	+	+	+	+	+	+	+	+
	0.74/Green	+	+	+	+	-	-	-	-	-	-	-	-	+	+
	0.76/Blue	+	+	+	+	-	+	+	+	+	+	+	+	+	+
	0.81/Blue	+	+	+	+	-	+	-	+	-	+	+	+	+	+
	0.93/Green-Blue	-	-	-	-	-	-	-	-	-	-	-	-	+	+

(+) Detected; (-) Not detected.

**Table 4:** Linearity, LOD, and LOQ data for the standards investigated.

Standards	Linearity Range (µg/mL)	r^2^	S *	Intercept	SD **	LOD (µg/mL)	LOQ (µg/mL)
*N*^1^,*N*^5^,*N*^10^-tricaffeoylspermidine	0.5–50	0.9986	17,251	15,374.333	331.482	0.058	0.192
Rutin		0.9996	7,345	427.920	126.484	0.052	0.172
Myricitrin		0.9999	9,531	1,833.133	176.365	0.056	0.185
Hyperoside		0.9997	11,143	3258.767	460.255	0.124	0.413
Isoquercitrin		0.9989	9,942	825.153	342.088	0.103	0.344
Quercitrin		0.9985	16,156	9,244.433	666.800	0.124	0.413
Quercetin		0.9997	31,160	23,892.333	154.500	0.015	0.050

* S: slope; ** SD: standard deviation of intercept.

**Table 5:** Intraday and interday precision data.

	Intraday Precision		Interday Precision	
Standards (5 µg/mL)	Average Concentration (µg/mL ± SD) (*n* = 3)	RSD (%) (*n* = 3)	Average Concentration (µg/mL ± SD) (*n* = 3)	RSD (%) (*n* = 3)
*N*^1^,*N*^5^,*N*^10^-tricaffeoylspermidine	4.914 ± 0.023	0.470	4.906 ± 0.025	0.512
	4.862 ± 0.026	0.528	4.991 ± 0.012	0.239
	4.906 ± 0.025	0.512		
Rutin	5.030 ± 0.045	0.891	5.104 ± 0.026	0.512
	5.002 ± 0.041	0.825	5.091 ± 0.018	0.348
	4.983 ± 0.018	0.358		
Myricitrin	4.822 ± 0.015	0.318	4.845 ± 0.033	0.678
	4.855 ± 0.024	0.489	5.062 ± 0.001	0.015
	4.905 ± 0.034	0.684		
Hyperoside	4.799 ± 0.027	0.573	4.929 ± 0.030	0.616
	4.838 ± 0.028	0.585	5.025 ± 0.016	0.325
	4.823 ± 0.013	0.279		
Isoquercitrin	5.191 ± 0.032	0.622	5.198 ± 0.032	0.615
	5.211 ± 0.026	0.501	5.284 ± 0.015	0.281
	5.178 ± 0.045	0.877		
Quercitrin	4.823 ± 0.012	0.245	4.813 ± 0.021	0.427
	4.754 ± 0.029	0.614	4.877 ± 0.012	0.253
	4.834 ± 0.005	0.103		
Quercetin	4.895 ± 0.003	0.069	4.904 ± 0.025	0.515
	4.880 ± 0.021	0.429	4.895 ± 0.015	0.298
	4.928 ± 0.013	0.256		

**Table 6:** Recovery of standards investigated.

Standards	Theoretical Value (µg/mL) (*n* = 3)	Amount Found (μg/mL ± SD) (*n* = 3)	Recovery (%) (*n* = 3)	RSD (%) (*n* = 3)
*N*^1^,*N*^5^,*N*^10^-tricaffeoylspermidine	3	2.816 ± 0.005	93.9	0.189
	6	5.786 ± 0.029	96.4	0.494
	12	11.814 ± 0.012	98.4	0.099
Rutin	3	3.094 ± 0.014	103.1	0.447
	6	5.902 ± 0.028	98.4	0.478
	12	12.404 ± 0.022	103.4	0.176
Myricitrin	3	2.866 ± 0.003	95.5	0.114
	6	5.821 ± 0.008	97.0	0.141
	12	11.879 ± 0.041	99.0	0.348
Hyperoside	3	2.904 ± 0.013	96.8	0.464
	6	6.075 ± 0.021	101.2	0.347
	12	12.351 ± 0.008	102.9	0.063
Isoquercitrin	3	2.947 ± 0.019	98.2	0.648
	6	6.067 ± 0.032	101.1	0.535
	12	12.139 ± 0.045	101.2	0.373
Quercitrin	3	3.161 ± 0.017	105.4	0.546
	6	6.191 ± 0.040	103.2	0.653
	12	12.242 ± 0.009	102.0	0.077
Quercetin	3	2.962 ± 0.005	98.7	0.172
	6	5.655 ± 0.008	94.2	0.142
	12	11.618 ± 0.015	96.8	0.126

**Table 7:** Contents of compounds investigated in bee pollen and androecia samples.

Samples	*N*^1^,*N*^5^,*N*^10^-tricaffeoylspermidine	Rutin	Myricitrin	Hyperoside	Isoquercitrin	Quercitrin	Quercetin
Bee Pollen	mg/g ± SD (*n* = 3)						
C1	40.96 ± 0.22 ^a^	N.d.	N.d.	N.d.	N.d.	N.d.	N.d.
C2	27.96 ± 0.14 ^b^	N.d.	N.d.	N.d.	N.d.	N.d.	N.d.
C3	25.57 ± 0.16 ^c^	N.d.	N.d.	N.d.	N.d.	N.d.	N.d.
S1	6.54 ± 0.02 ^g^	N.d.	N.d.	N.d.	N.d.	N.d.	N.d.
S2	21.24 ± 0.07 ^d^	N.d.	N.d.	N.d.	N.d.	N.d.	N.d.
S3	4.86 ± 0.01 ^k^	N.d.	N.d.	N.d.	N.d.	N.d.	N.d.
S4	5.54 ± 0.04 ^i^	N.d.	N.d.	N.d.	N.d.	N.d.	N.d.
S5	6.20 ± 0.03 ^h^	N.d.	N.d.	N.d.	N.d.	N.d.	N.d.
S6	8.56 ± 0.11 ^f^	N.d.	N.d.	N.d.	N.d.	N.d.	N.d.
S7	14.17 ± 0.09 ^e^	N.d.	N.d.	N.d.	N.d.	N.d.	N.d.
Q1	3.15 ± 0.01 ^m^	0.95 ± 0.02 ^a^	N.d.	N.d.	N.d.	N.d.	N.d.
**Androecia**	**mg/g ± SD (*n* = 3)**						
*Castanea sativa*	6.19 ± 0.05 ^h^	N.d.	16.14 ± 0.08	4.14 ± 0.07 ^b^	8.76 ± 0.13 ^b^	2.55 ± 0.02	N.d.
*Salix alba*	3.48 ± 0.01 ^l^	0.59 ± 0.01 ^b^	N.d.	N.d.	1.64 ± 0.03 ^c^	N.d.	N.d.
*Quercus pubescens*	5.19 ± 0.05 ^j^	0.47 ± 0.06 ^c^	N.d.	6.96 ± 0.06 ^a^	10.79 ± 0.04 ^a^	N.d.	2.46 ± 0.02

N.d.: Not detected. Different letters “a–m” in the same column indicate statistically significant differences (*p* ≤ 0.05).

**Table 8:** *In vitro* antioxidant activity of *N*^1^,*N*^5^,*N*^10^-tricaffeoylspermidine, bee pollen, and androecia samples determined by DPPH, FRAP, ABTS, and CUPRAC assays.

Standard and Samples	DPPH	FRAP	ABTS	CUPRAC
	mg TE/g (*n* = 3)			
*N*^1^,*N*^5^,*N*^10^-tricaffeoylspermidine	966.28 ± 28.72 ^a^	768.97 ± 11.44 ^a^	1047.65 ± 35.65 ^a^	610.35 ± 15.69 ^a^
	**mg TE/g extract (*n* = 3)**			
**Bee Pollen**				
C1	42.04 ± 0.79 ^cde^	28.34 ± 1.33 ^d^	141.94 ± 1.08 ^cd^	47.94 ± 2.27 ^cd^
C2	46.37 ± 0.55 ^cd^	38.75 ± 0.66 ^c^	154.65 ± 2.06 ^c^	45.09 ± 1.52 ^d^
C3	41.76 ± 0.60 ^cde^	18.40 ± 0.28 ^e^	125.79 ± 1.15 ^d^	41.54 ± 1.28 ^d^
S1	18.09 ± 0.68 ^f^	13.81 ± 0.12 ^ef^	69.39 ± 0.63 ^ef^	24.08 ± 0.33 ^e^
S2	23.28 ± 0.37 ^ef^	14.90 ± 0.59 ^e^	77.33 ± 0.56 ^e^	24.67 ± 1.00 ^e^
S3	16.44 ± 0.35 ^f^	12.05 ± 0.35 ^ef^	63.63 ± 1.27 ^ef^	25.46 ± 0.65 ^e^
S4	8.88 ± 0.60 ^f^	5.35 ± 0.12 ^f^	47.66 ± 1.56 ^f^	21.33 ± 0.80 ^e^
S5	12.46 ± 0.28 ^f^	10.01 ± 0.88 ^ef^	60.46 ± 0.34 ^ef^	23.50 ± 0.73 ^e^
S6	16.69 ± 0.38 ^f^	11.76 ± 0.34 ^ef^	66.25 ± 0.95 ^ef^	24.10 ± 0.22 ^e^
S7	24.59 ± 0.62 ^def^	12.18 ± 0.60 ^ef^	71.79 ± 0.58 ^ef^	26.03 ± 1.14 ^e^
Q1	16.41 ± 1.03 ^f^	10.37 ± 0.21 ^ef^	63.53 ± 0.80 ^ef^	27.05 ± 0.38 ^e^
**Androecia**				
*Castanea sativa*	165.98 ± 3.14 ^b^	111.92 ± 1.52 ^b^	309.08 ± 3.91 ^b^	177.41 ± 2.54 ^b^
*Salix alba*	25.13 ± 0.20 ^def^	27.79 ± 0.75 ^d^	120.98 ± 1.01 ^d^	47.99 ± 1.21 ^cd^
*Quercus pubescens*	51.56 ± 1.21 ^c^	39.22 ± 0.67 ^c^	147.05 ± 1.14 ^cd^	60.47 ± 1.58 ^c^

Different letters “a–f” in the same column indicate statistically significant differences (*p* ≤ 0.05).

**Table 9:** XO inhibitory activity of allopurinol, *N*^1^,*N*^5^,*N*^10^-tricaffeoylspermidine, bee pollen, and androecia samples.

Standards and Samples	XO Inhibitory Activity
	IC_50_ (µg/mL ± SD) (*n* = 3)
Allopurinol (Positive Control)	3.07 ± 0.05 ^a^
*N*^1^,*N*^5^,*N*^10^-tricaffeoylspermidine	2.98 ± 0.19 ^a^
**Bee Pollen**	
C1	26.18 ± 0.99 ^de^
C2	29.30 ± 1.94 ^ef^
C3	31.52 ± 3.48 ^fg^
S1	54.29 ± 0.70 ^j^
S2	34.36 ± 1.72 ^g^
S3	63.77 ± 2.39 ^kl^
S4	62.79 ± 0.91 ^k^
S5	49.60 ± 2.19 ^ij^
S6	45.05 ± 0.41 ^hi^
S7	40.78 ± 1.80 ^h^
Q1	67.75 ± 2.44 ^l^
**Androecia**	
*Castanea sativa*	15.97 ± 0.21 ^b^
*Salix alba*	23.74 ± 0.93 ^cd^
*Quercus pubescens*	20.83 ± 0.68 ^bc^

Different letters “a–l” in the same column indicate statistically significant differences (*p* ≤ 0.05).

## Data Availability

The data presented in this study are available upon request from the corresponding authors.

## Supplementary Materials

The following supporting information can be downloaded at https://www.mdpi.com/article/10.3390/antiox14010040/s1: Figure S1: HRMS spectrum of *N*^1^,*N*^5^,*N*^10^-tricaffeoylspermidine; Figure S2: 1H NMR spectrum of *N*^1^,*N*^5^,*N*^10^-tricaffeoylspermidine (400 MHz, DMSO); Figure S3: ^13^C NMR spectrum of *N*^1^,*N*^5^,*N*^10^-tricaffeoylspermidine (100 MHz, DMSO); Figure S4: Influence of the type of stationary phase (HPTLC silica gel F_254_ and HPTLC NH_2_ F_254S_ plates) and time on the detection of XO inhibitors. Allopurinol (positive control, track 1) and *N*^1^,*N*^5^,*N*^10^-tricaffeoylspermidine (track 2) were applied on the plates after development with ethyl acetate–formic acid–water (35:4:4, *v*/*v*/*v*). Plates were documented before HPTLC-XO assay (at 254 and 366 nm) and after HPTLC-XO assay (under white light) at different time intervals (0–120 min).
