# Supplementary material for: Phytochemical and Bioactivity Evaluation of Bee Pollen and Androecia of Castanea, Salix, and Quercus Species
Source: Antioxidants (Basel). 2024 Dec 31;14(1):40. doi: 10.3390/antiox14010040 (PMC11760459; doi:10.3390/antiox14010040)
Supplement: Supplementary file 1 [file antioxidants-14-00040-s001.zip › antioxidants-3392001-supplementary.pdf]

SUPPLEMENTARY MATERIAL

Phytochemical and Bioactivity Evaluation of Bee Pollen and Androecia of *Castanea*, *Salix*, and *Quercus* species

Nisa Beril Sen [1], Irena Vovk [2,*,†], Hasan Kırmızıbekmez [1] and Etil Guzelmeric [1,*,†]

[1] Department of Pharmacognosy, Faculty of Pharmacy, Yeditepe University, Kayisdagi Cad., Atasehir, 34755, Istanbul, Türkiye; nisaberil.sen@yeditepe.edu.tr (N.B.S.), hkirmizibekmez@yeditepe.edu.tr (H.K.), etil.ariburnu@yeditepe.edu.tr (E.G.)

[2] Laboratory for Food Chemistry, National Institute of Chemistry, Hajdrihova 19, SI-1000 Ljubljana, Slovenia; irena.vovk@ki.si (I.V.)

Correspondence: irena.vovk@ki.si (I.V.); Tel.: +386-1476-0341; etil.ariburnu@yeditepe.edu.tr (E.G.); Tel.: +90-216-578-0558

[†]These authors have contributed equally to this work.

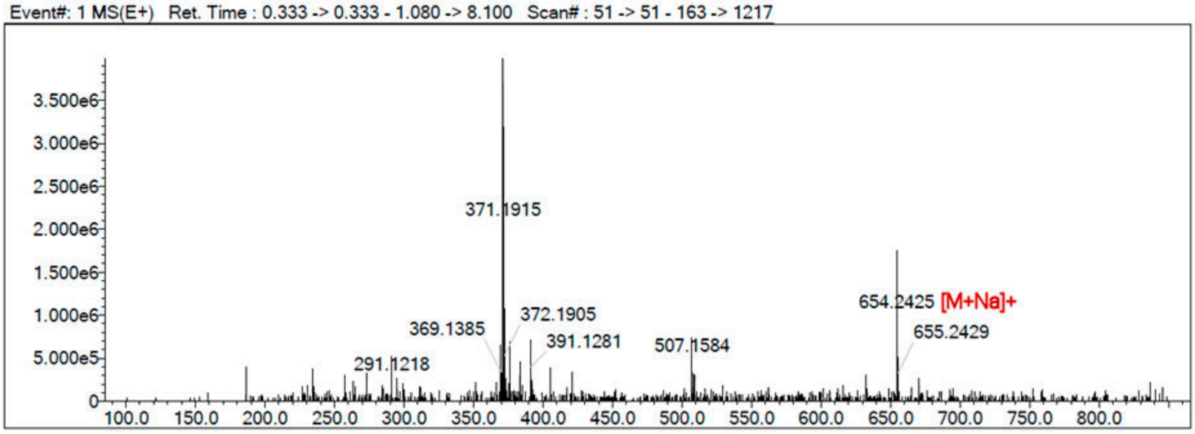

Rank	Score	Formula (M)	Ion	Meas. m/z	Pred. m/z	Df. (mDa)	Df. (ppm)	Iso	DBE
1	37.64	C34 H37 N3 O9	[M+Na]+	654.2425	654.2422	0.3	0.46	37.64	18.0

Figure S1. HRMS spectrum of *N*[1],*N*[5],*N*[10]-tricaffeoylspermidine.

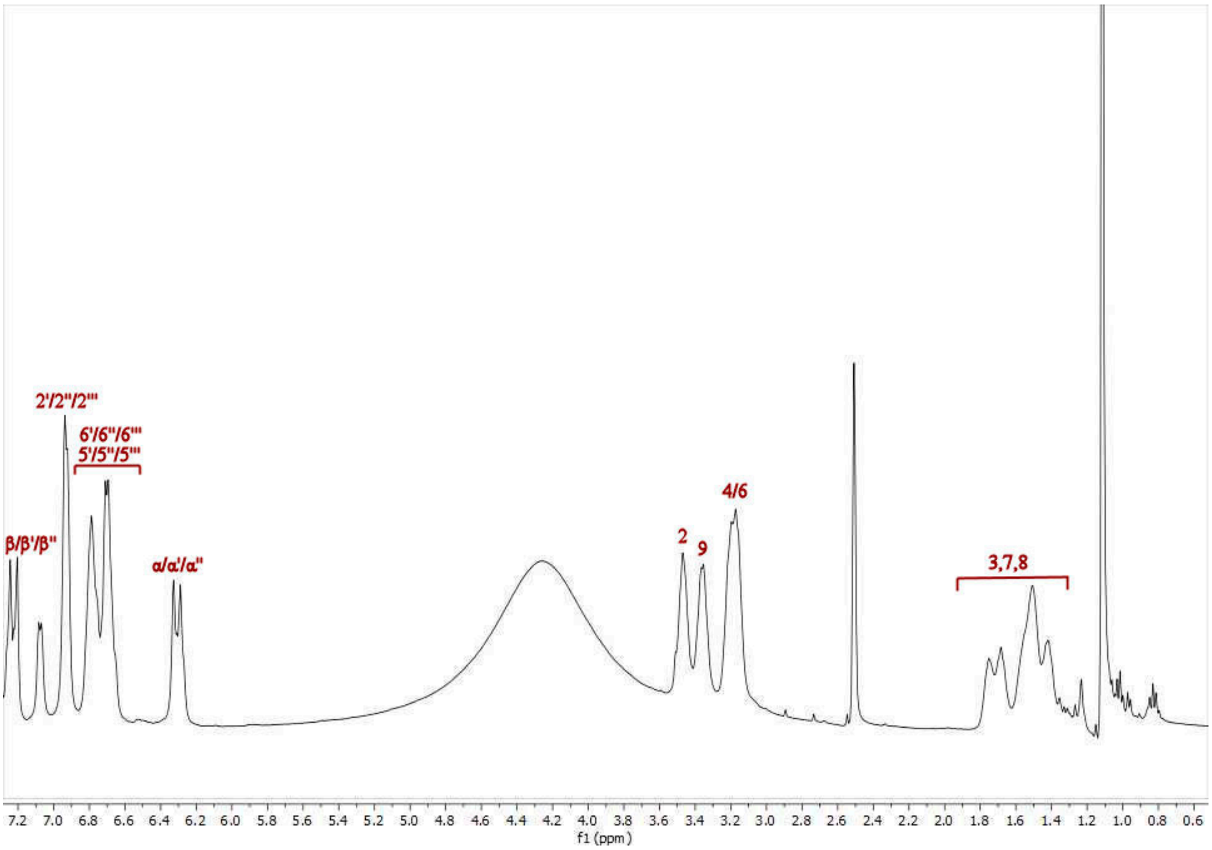

Figure S2. ^{1}H NMR spectrum of *N*^{1},*N*^{5},*N*^{10}-tricaffeoylspermidine (400 MHz, DMSO).

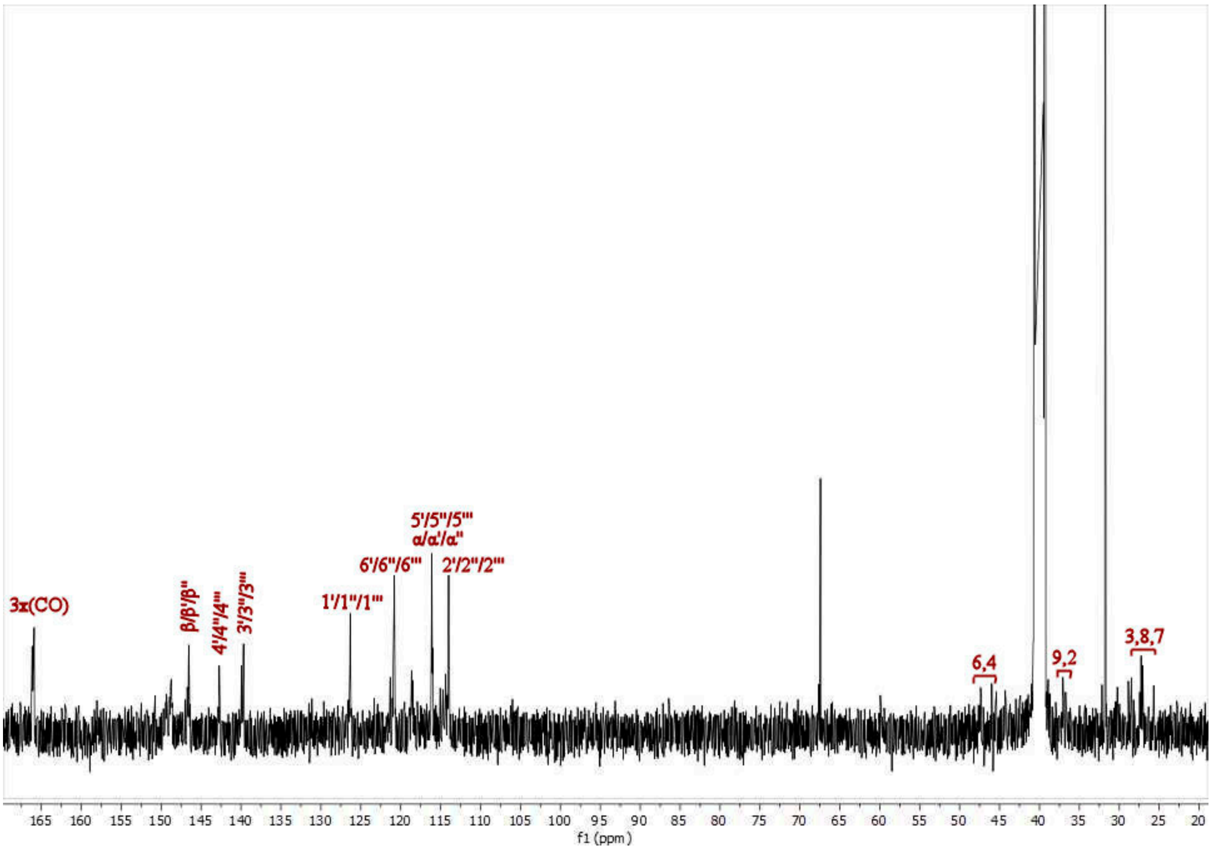

Figure S3. ^{13}C NMR spectrum of N^1,N^5,N^{10}-tricaffeoylspermidine (100 MHz, DMSO).

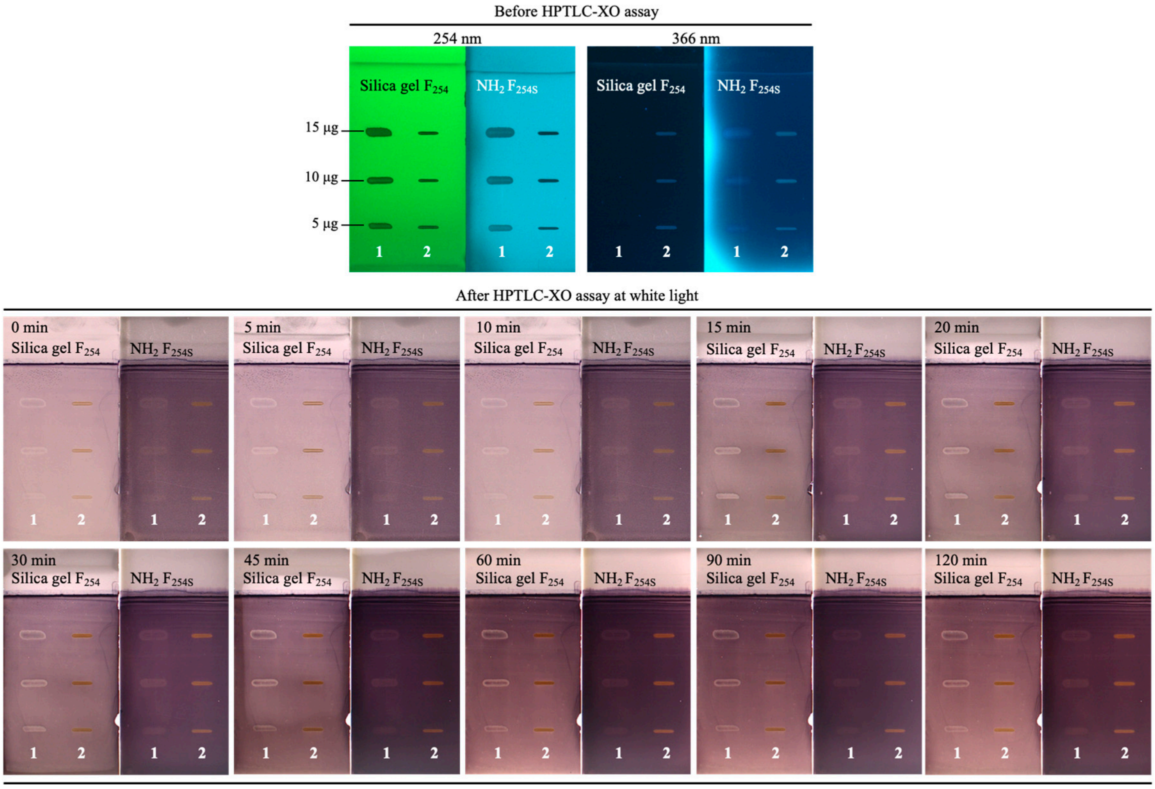

Figure S4. Influence of the type of stationary phase (HPTLC silica gel F_{254} and HPTLC NH_2 F_{254S} plates) and time on the detection of XO inhibitors. Allopurinol (positive control, track 1) and N^1,N^5,N^{10}-tricaffeoylspermidine (track 2) were applied on the plates after development with ethyl acetate–formic acid–water (35:4:4, *v*/*v*/*v*). Plates were documented before HPTLC-XO assay (at 254 and 366 nm) and after HPTLC-XO assay (at white light) at different time intervals (0-120 min).
